# Self-management interventions for skin care in people with a spinal cord injury: part 1—a systematic review of intervention content and effectiveness

**DOI:** 10.1038/s41393-018-0138-3

**Published:** 2018-05-25

**Authors:** Justine S. Baron, Katrina J. Sullivan, Jillian M. Swaine, Arlene Aspinall, Susan Jaglal, Justin Presseau, Barry White, Dalton Wolfe, Jeremy M. Grimshaw

**Affiliations:** 1 Clinical Epidemiology Program, Ottawa Hospital Research Institute, Ottawa, ON Canada; 2 Faculty of Health and Medical Sciences, University of Western Australia, Perth, WA Australia; 3 Institute for Health Research, University of Notre Dame Australia, Fremantle, WA Australia; 4 Rick Hansen Institute, Vancouver, BC Canada; 5 Vancouver General Hospital, Vancouver, BC Canada; 6 Department of Physical Therapy, University of Toronto, Toronto, ON Canada; 7 Toronto Rehabilitation Institute, Toronto, ON Canada; 8 School of Epidemiology and Public Health, University of Ottawa, Ottawa, ON Canada; 9 Parkwood Institute Research, Lawson Health Research Institute, London, ON Canada; 10 University of Western Ontario, London, ON Canada; 11 Department of Medicine, University of Ottawa, Ottawa, ON Canada

## Abstract

**Study design:**

Systematic review.

**Objectives:**

To review the content and effectiveness of skin care self-management interventions for people with SCI.

**Setting:**

International.

**Methods:**

We searched electronic bibliographic databases, trial registers, and relevant reference lists. Eligibility criteria for the reviews of intervention content and effectiveness were identical with the exception of study design. The review of intervention content included non-randomized and randomized controlled trials (RCTs). The review of effectiveness included RCTs. A Behavior Change Technique (BCT) taxonomy of 93 BCTs was used to code intervention content. Intervention effects on outcomes of interest are summarized descriptively. Effect sizes were calculated, and the Cochrane risk of bias tool applied.

**Results:**

In all, 15 studies testing 17 interventions were included in the review of intervention content. Interventions in these studies included 28 BCTs. The most common were “instructions on how to perform behavior” (16 interventions), “credible source” (12 interventions), and “social support (unspecified)” (9 interventions). Ten RCTs were included in the review of intervention effectiveness and they measured knowledge, self-efficacy, and skills relating to skin care/pressure ulcer (PU) prevention, skin care behaviors, skin status (PU prevalence, severity, and time to PU), and health-care utilization for skin problems. Evidence to support intervention effects on these outcomes was limited, particularly for clinical outcomes. Risk of bias assessments was often inconclusive due to poor reporting.

**Conclusions:**

There is potential to design SCI skin care interventions that include currently untested BCTs. Further research and better consistency in outcome measurements and reporting are required to synthesize evidence on effectiveness.

## Introduction

Pressure ulcers (PUs; or pressure injuries) are among the commonest secondary complications affecting people with a spinal cord injury (SCI) living in the community. Period prevalence over 3 months has been found to be as high as 34.7% [1]. PUs lead to high rates of health-care utilization following discharge from SCI rehabilitation, and one PU adds an average of $18,758 to hospital admission costs in Canada [2]. They result in significant social (e.g., isolation) and financial (e.g., unemployment) limitations, as well as psychological difficulties (e.g., negative emotions) [3].

One way to help prevent PUs in the SCI community is by influencing modifiable risk factors, such as patients’ self-care behaviors. Preventive skin care is less than optimal in the SCI community, with one study suggesting that 29.9% of PUs are associated with self-care behaviors [4]. Some skin care behaviors are commonly performed by people with SCI (e.g., skin care in case of incontinence and examination of the cause of PUs). Others such as daily skin checks and pressure relief are performed by 50% or fewer [5]. Similarly, less than 5% of people with SCI adhere to dietary recommendations [6], 37% are inactive [6], and 30% delay visiting a physician after detecting a PU [7].

Self-management interventions for people with SCI have been designed to reduce non-adherence to preventive skin care. Three reviews [8–10] have synthesized this work, but they focus only on educational interventions, technology-based interventions, or behavioral and educational interventions with a primary focus on PU prevention. None of these reviews has used a systematic approach to describe intervention content. Systematically describing intervention content is an important step in building a cumulative science [11]. The aims of this study were to: (1) review the active ingredients in self-management interventions for skin care in SCI and (2) review the effectiveness of these same interventions. A separate publication [12] reports on theory use and reporting quality.

## Methods

We published a protocol [13] prior to undertaking this review. Common search and study selection procedures were used to address aims 1 and 2. Eligibility criteria for these aims were also identical, with the exception of study design. A greater number of study designs were eligible to address aim 1 as this work was performed to better understand the content of the interventions designed in this area of research. In contrast, the focus on effectiveness in aim 2 resulted in restricting study designs to randomized controlled trials (RCTs).

### Search strategy

#### Bibliographic databases

A MEDLINE search strategy was designed to include search terms on SCI, self-management, and skin care. The MEDLINE search strategy was peer-reviewed by an independent librarian using the Peer-Review of Electronic Search Strategy checklist [14]. In consultation with the librarian, it was adapted for use in four other large electronic databases (Embase, PsycINFO, CENTRAL, and CINAHL). Smaller databases (REHABDATA, CIRRIE, PeDro, and ERIC) were searched using keywords and subject headings if available. All search strategies were run on 23 February 2016 and are presented in Supplementary Information 1.

#### Additional data sources

Relevant posters, abstracts, and conference proceedings identified via the electronic bibliographic database search were used to search for papers. In addition, reference lists of relevant published protocols, systematic reviews, and of the final list of included studies were hand-searched. Authors were contacted for further information and publications. Authors announcing a forthcoming publication were recontacted in March 2017 to identify accepted or published papers.

Trial registers (World Health Organization International Clinical Trials Registry and Meta-Register of Controlled Trials) were searched on 21 June 2016 using keywords (see Supplementary Information 1). Publications related to relevant studies were searched. If none were found, principal investigators were contacted for information and publications.

### Eligibility criteria

To address aim 1 (review of the content of skin care self-management interventions for people with SCI), RCTs and non-randomized trials with a control group receiving standard care and published in English were included. Studies must have primarily involved people with SCI (representing 50% or more of the sample). Included studies tested interventions that addressed, at least in part, skin care self-management capabilities related to PU prevention. Given that multiple behaviors are often addressed to varying degrees in SCI self-management interventions, studies needed to include measurement of at least one of the following outcomes of interest: mediators of skin care behaviors (e.g., self-efficacy or skills, and knowledge relating to skin care or PU prevention), skin care behaviors, and PU-related clinical outcomes (e.g., PU prevalence, incidence, re-occurrence, or severity). Studies with a primary focus on PU treatment were excluded, as were studies targeting more lifestyle-related behaviors (e.g., improving nutritional intake or physical activity and smoking cessation) that can affect physiological indicators of skin health. No exclusion criteria were applied with respect to intervention delivery setting (e.g., inpatient, outpatient, and community), length of follow-up, or publication date.

The same eligibility criteria were applied to address aim 2 (review on effectiveness), with the exception of study design that was restricted to RCTs.

### Study selection

Two reviewers (J.S.B. and J.M.S.) independently screened titles and abstracts of bibliographic database search results. Articles not meeting inclusion criteria were removed. Full texts of remaining publications were then independently reviewed.

J.S.B. was responsible for the identification of potentially relevant papers using additional data sources. J.M.S. screened papers identified as potentially relevant based on their titles and abstracts. Both reviewers then independently screened full texts.

Discrepancies in screening outcomes were discussed until consensus was reached. A third reviewer (J.M.G.) was consulted in case of disagreement.

### Data extraction

A data extraction spreadsheet was designed in Excel to capture general information, study characteristics, participants, intervention characteristics, measurements, data analyses, and intervention effects (see protocol [13]). It was piloted on two papers reporting SCI behavioral interventions and ineligible for inclusion in this review.

Two reviewers (J.S.B. and K.J.S.) independently extracted data. Discrepancies were discussed until consensus was reached, or a third party was consulted (J.M.G.).

### Aim 1: Review of intervention content (RCTs and non-RCTs)

Data extraction for intervention content was based on intervention and control group treatment descriptions in eligible papers, and in any related papers and intervention materials located by reviewers (e.g., cited in text) or during correspondence with authors. These materials could include published protocols, intervention pilots, intervention development papers, and any unpublished document supporting intervention delivery (e.g., intervention manuals, scripts, and PowerPoint slides). Intervention components delivered to SCI participants were coded into behavior change techniques (BCTs) [11], defined as the smallest observable and replicable components of behavioral interventions designed to bring about change. The BCT taxonomy [11] includes 93 BCTs (see Supplementary Information 2). It comes with online training, which both coders completed. Our protocol suggested that we would also use a second, broader taxonomy of self-management components [15]. Results using this broader taxonomy were not considered to add value to the more precise BCT taxonomy codes and have, therefore, not been reported here (available upon request).

### Aim 2: Review of intervention effectiveness (RCTs only)

We examined effectiveness of interventions tested in RCTs, and focused on extracting data for time points where intervention and control group participants were assessed. It was anticipated that a meta-analysis would not be feasible, given our preliminary knowledge of the varied outcome measures in SCI self-management studies. Intervention effects are described, classified by outcome type (mediators of skin care, skin care behaviors, and clinical outcomes) but are not statistically pooled.

We aimed to present effect sizes and 95% confidence intervals (CIs) where sufficient data were available and for studies with samples larger than 10 participants. Hedges’ *g* [16] effect size was used for continuous outcomes and odds ratio (OR) was used for categorical data. Hedges’ *g* is interpreted the same way as Cohen’s *d* (0.2 = small, 0.5 = medium, and 0.8 = large). Data pertaining to the longest follow-up were used for these effect size calculations. We report continuous outcomes as a percentage change from baseline and as a mean score at follow-up if both were provided, but the effect size was computed using mean scores. When scale and individual item scores were available for the same outcome, the former were used for effect size calculations. Authors were contacted when outcome data were incomplete or unclear.

To complement our assessment of effectiveness with one of study quality, two reviewers (J.S.B. and K.J.S.) independently assessed risk of bias using the Cochrane tool [17]. This tool focuses on random sequence generation, allocation concealment, blinding of study personnel and outcome assessors, incomplete outcome data, selective reporting, and other sources of bias (rated as “low risk”, “high risk”, or “unclear risk” of bias). The domain blinding of outcome assessors was split into two items, one for mediators of behavior and the second for PU-related clinical outcomes, and studies that did not include these outcomes were rated as “Not Applicable”. Disagreements between reviewers were resolved by discussion. A third author (J.G.) was consulted for arbitration.

## Results

**Fig. 1:**
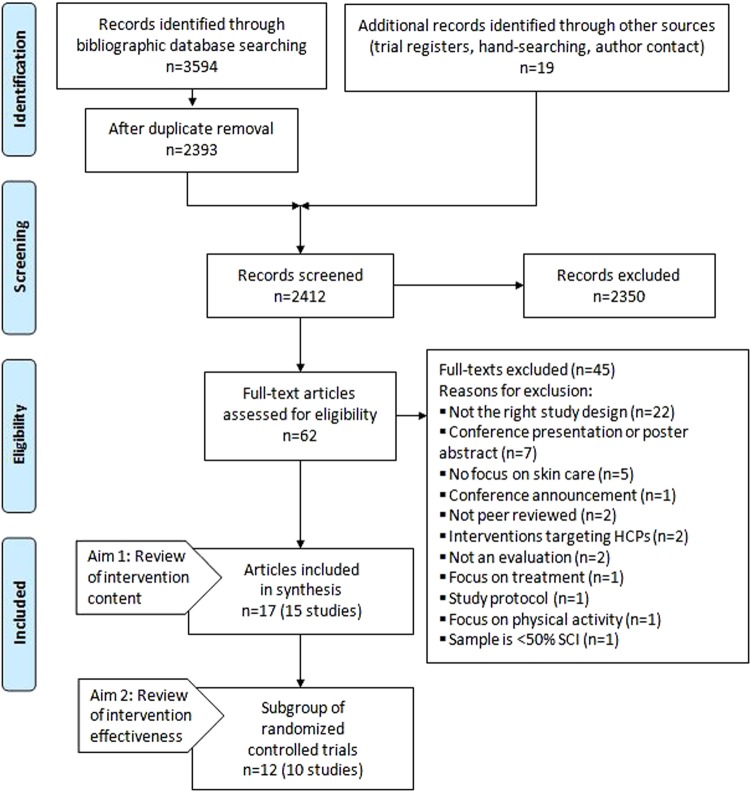
PRISMA flow diagram. Systematic review of skin care self-management interventions for people with SCI

Figure 1 is a PRISMA flow diagram. Electronic bibliographic databases and additional data source searching returned 2412 papers. Following title and abstract screening, 62 were screened in full text. Seventeen were eligible for the review on intervention content (aim 1). A list of excluded studies is available in Supplementary Information 3. These 17 papers described 15 studies, 10 of which were RCTs and therefore included in the review on intervention effectiveness (aim 2). Publications used to maximize data extraction included a published protocol [18], intervention development or pilot test publications [19–21], and one erratum [22].

### Aim 1: Review of intervention content (RCTs and non-RCTs)

The 15 studies reviewed tested 17 different interventions. Fifteen control groups received standard care consisting in very limited or no contact with health-care professionals (HCPs) or intervention deliverers (10 studies [23–33]), and minimal education (five studies [22, 34–38]). A description of intervention and control group treatments is in Table 1.

**Table 1:** Main study characteristics for randomized controlled trials and non-randomized study designs

Study, country, design and sample size	Recruitment pool, Sample characteristics	Purpose of interventions	Timing of intervention	Length of intervention	Description of treatment groups and coded BCTs
**RCTs**					
Garber [22], Rintala [36], United States, RCT^a^ I: *n* = 20C: *n* = 21	*Pool*: Inpatients *Sample*: Men with SCI and MS admitted to a medical center for Veterans for surgery to repair stage III or IV pelvic PU	To increase knowledge about PUs with the ultimate goal of delaying or preventing recurrence	During inpatient stay and after discharge	2 Years	*I: Enhanced education* *+* *structured phone follow-up*: 4 × 1 h of personalized education delivered over 2 weeks in hospital. Included modules on PU etiology, prevention strategies, nutrition, and pressure-relief support surfaces + monthly phone calls after discharge for 2 years or until recurrence of a PU to review behaviors and remind participants of those not performed. *BCTs*: Biofeedback, social support (unspecified), Instructions on how to perform behavior, information about health consequences, credible source *C: Standard inpatient program education*: 1–2 h individualized educational session on general prevention strategies (e.g., nutrition, smoking cessation, skin inspection, and care). Structured follow-up calls for data collection only. *BCTs*: social support (unspecified), credible source, and biofeedback
Guihan [34], United States, RCT I: *n* = 72C: *n* = 72	*Pool*: Inpatients *Sample*: Individuals with recent SCI (≥6 months post injury) admitted for treatment of a stage III or IV pelvic ulcer	To improve skin-protective behaviors and prevent skin worsening	Starting at discharge or within 2 years from discharge	6 Months	*Prior to randomization*^b^: Education in knowledge areas not conforming to guidelines, using a PU consumer guide. Standardized treatment components included seating evaluation (including pressure mapping, education on safe/effective transfers, and PU prevention strategies), nutritional assessment, skin care behavior demonstrations (pressure relief and skin checks), depression, substance abuse, bowel/bladder incontinence, and assessment of personal-care assistance *I: Self-management (SM) sessions* *+* *motivational interviewing (MI):* seven SM conference call sessions (45–60 min) on guideline-based skin care; problem solving and self-monitoring skills; community resource utilization; relaxation and mood management skills; relationships with providers; and development of action plans. Following group sessions, MI delivered in eight phone calls over 24 weeks to elicit change talk and commitment language having to do with improving skin care behaviors *BCTs:* Goal setting (behavior), problem solving, action planning, feedback on behavior, biofeedback, social support (unspecified), instruction on how to perform behavior, credible source, body changes, and self-talk *C: SM sessions without skills straining:* Treatment equivalent to the SM intervention above in terms of number and timing of sessions. Emphasis on teaching and advice giving while barring the active ingredients of skills training and MI *BCTs*: Instructions on how to perform behavior, biofeedback
Phillips [29], United States, RCT I 1: *n* = 36 I 2: *n* = 36C: *n* = 39	*Pool*: Outpatients *Sample*: Newly injured SCI patients discharged back to the community	To reduce the incidence of secondary conditions (including PUs)	Starting at discharge or within 2 years from discharge	9 Weeks	*I1: Video-based telehealth:* Individual, pre-scheduled, educational rehabilitation sessions (30–40 min) 1 × /week for 5 weeks, then once every 2 weeks for 1 month. Included structured review of skin care, nutrition, bowel and bladder routines, psychosocial issues, and discussion of any equipment needs + referrals (e.g., mental health, physical therapists) if necessary. *BCTs*: Social support (unspecified), instruction on how to perform the behavior, credible source *I2: Phone-based telehealth:* Same content as above but mode of delivery is phone-based *BCTs*: Social support (unspecified), instruction on how to perform the behavior, credible source *C: Standard care:* Patients encouraged to call rehabilitation center helpline if/when in need of assistance prior to the 2-month post-discharge visit *BCTs*: Social support (unspecified)
Houlihan [25], Mercier [33], United States, Pilot RCT I: *n* = 71C: *n* = 71	*Pool*: Outpatients *Sample*: Individuals with SCI or MS using a wheelchair for at least 6 h/day without a stage III PU	To reduce the prevalence and severity of secondary complications (including PUs) and improve access to health care	During life in community	6 Months	*I: ‘*”*Carecall*” *Interactive Voice Response (IVR) system*: Weekly calls for 3 months, then biweekly calls for 3 months. Combination of patient education, cognitive behavioral interventions, screening and referrals, and alerts to a nurse for direct non-emergent phone follow-up. Topics: skin care, depression and wellness, and health-care utilization. Content featured audio vignettes from patients and health-care professionals, and was personalized to patients’ previous responses. Patients received Carecall resource book (see below) *BCTs*: Problem solving, social support (unspecified), social support (practical), instruction on how to perform the behavior, information about health consequences, salience of consequences, prompts/cues, habit formation, credible source, material incentive (behavior), social reward, reduce negative emotions, body changes, and framing/reframing *C: Standard care including CareCall resource book:* Local and informational resources for topics like medical supplies, mental and physical health providers, and personal-care assistants *BCTs*: Social support (unspecified)
Hossain (2016), India, Pragmatic RCT I: *n* = 15C: *n* = 15	*Pool*: Outpatients *Sample*: Individuals with recent SCI (≤2 years) who require a wheelchair for daily mobility	To reduce mortality and improve quality of life following discharge	Starting at discharge or within 2 years from discharge	2 Years	*I: Phone-based monitoring and support:* Telephone contact (every 2 weeks during year 1, and monthly during year 2) + 3 home visits. Intervention deliverers (health-care professional) reviewed complications and provided advice/support. During complications, phone calls were more regular and help was provided to source local support, appropriate medical, and nursing care or hospitalization. Up to AUS $80 (study funder in Australia) provided to purchase care or equipment *BCTs*: Problem solving, social support (unspecified), social support (practical), instruction on how to perform the behavior, credible source *C: Standard care:* one phone call and optional home visit *BCTs*: Social support (unspecified), credible source
Worobey [38], United States, RCT I: *n* = 55C: *n* = 59	*Pool*: Outpatients *Sample*: People with non-progressive SCI using a manual wheelchair as a primary means of mobility	To improve wheelchair skills and achievement of individually set goals	During life in community	6 Weeks	*I: Wheelchair skills training program including pressure relief:* Six weekly 90 min classes involving hands-on demonstrations and practice of selected wheelchair skills (primarily related to mobility with one on pressure relief) *BCTs*: Goal setting (behavior), feedback on behavior, biofeedback, social support (unspecified), instruction on how to perform the behavior, information about social and environmental consequences, demonstration of the behavior, prompts/cues, behavioral practice/rehearsal, graded tasks, credible source, mental rehearsal of successful performance *C: Two 1-h general education classes:* Group classes scheduled 1–3 weeks apart. Class topics were aging with a SCI, weight management, and nutrition *BCTs*: Credible source
Best [23], Canada, Pilot RCT I: *n* = 16C: *n* = 12	*Pool*: Outpatients *Sample*: Manual wheelchair users (2 h/day or more)included people with SCI, cerebral palsy, stroke, Parkinson's disease, amputation	To improve manual wheelchair skills capacity and performance	During life in community	3–6 Weeks	*I: Wheelchair skills training program (Wheelsee) including pressure relief:* Six 90-min sessions (1–2 sessions/week) delivered to pairs to work on patient-identified wheelchair skills (including pressure relief) and less tangible skills (discussions and role play to improve knowledge, problem solving, advocacy, managing social situations, controlling emotions) *BCTs*: Goal setting (behavior), problem solving, feedback on behavior, biofeedback, social support (unspecified), social support (practical), instruction on how to perform the behavior, information about social and environmental consequences, demonstration of the behavior, social comparison, prompts/cues, behavioral practice/rehearsal, graded tasks, credible source, framing/reframing, verbal persuasion about capability, and mental rehearsal of successful performance *C: No training received or contact made with patients* *BCTs*: None coded.
Ozturk [27], Turkey, RCT I: *n* = 17C: *n* = 15	*Pool*: Outpatients *Sample*: Manual wheelchair users. Included people with SCI, spinal cord disorders, congenital hip dislocation, meningitis, total hip replacement, osteoarthritis, stroke, amputations, postpolio, and cerebral palsy	To improve wheelchair skills performance and safety	During life in community	4 Weeks	*I: Wheelchair skills training program including pressure relief:* 45-mn sessions (3 × per week) focusing on the wheelchair skills unsuccessfully completed at baseline (including pressure relief), and starting with basic skills to move toward advanced skills. Also included home visits during which trainer observed patients’ environmental and living conditions in order to individualize the training. *BCTs*: Goal setting (behavior), problem solving, feedback on behavior, biofeedback, social support (unspecified), instruction on how to perform the behavior, information about social and environmental consequences, demonstration of the behavior, prompts/cues, behavioral practice/rehearsal, graded tasks, credible source, and mental rehearsal of successful performance *C: No training received or contacts made with patients* BCTs: None coded
Rowland [31], United States, RCT I: *n* = NR C: *n* = NR Total *N* = 71	*Pool*: Outpatients *Sample*: Individuals with recent (6–18 months) traumatic SCI without stage IV pressures ulcer at start of study	To increase health behaviors associated with the reduction of secondary conditions including PUs	Starting at discharge or within 2 years from discharge	1.5–2 h	*I: Survey-based risk assessment and feedback (1.5–2* *h session):* Patients completed computer-based surveys including behavioral and knowledge questions relating to secondary condition risk factors. Patients’ responses were combined using an algorithm to generate an individualized risk score (1–5) for each complication. Participants with moderate to high risk scores (1.67 and above) met individually with a consultant to discuss preventive actions. Site consultants based their recommendations on the information included in a series of Secondary Condition Booklets provided by study coordinators *BCTs*: Instruction on how to perform the behavior, salience of consequences, credible source *C: Same computer-based survey as above without feedback:* Feedback was provided after the end of the study BCTs: None coded
Rottkamp [30], United States, RCT I: *n* = 5C: *n* = 5	*Pool*: Inpatients *Sample*: Patients with SCI and the ability to move upper extremities through partial or complete range of motion	To improve body-positioning performance both in terms of changes in body positioning and participant independence in body positioning	During inpatient stay only	4 Weeks	*I: Body-positioning training:* Body-positioning approach (and goals) presented to patients, followed by 6–12 × /week training sessions (10–60 mn each). Included the review of a diagrammed illustration of body positions, followed by demonstration, practice, and repetition of body-positioning changes (included manual, verbal, gestural, and written cues) until successful completion with minimal assistance. A personalized daily body-positioning schedule was placed within patients’ reach. Patients were observed, instructed, and reinforced at intervals to ensure the schedule was followed *BCTs*: Feedback on behavior, social support (practical), instruction on how to perform the behavior, demonstration of the behavior, prompts/cues, behavioral practice/rehearsal, and social reward *C: Standard care:* Customary body-positioning nursing care, i.e., passive participation in body positioning with lifting performed by the nursing staff. changes of position at standard intervals based on ward routines. Choice of body positions guided by patient preferences rather than positions prescribed. Observable teaching in body positioning took the form of verbal instructions *BCTs*: Instructions on how to perform behavior
**Non- RCTs**					
Scotzin [37], United States, Non-equivalent control group I: *n* = 22C: *n* = 20	*Pool*: Inpatients *Sample*: Patients with paraplegia or quadriplegia (people with traumatic SCI and spinal cord disease)	To improve patients’ motivation to perform skin care	During inpatient stay only	Unclear	*I: Motivational education program* “*Don’t Just Sit There*”: six session program based on the Multidimensional Model of Motivation *BCTs*: None coded *C: Standard inpatient education program*: 10 sessions including information on skin care and other topics of SCI management *BCTs*: None coded
Schopp [32], United States, Non-randomized, two group trial People with a SCI: I: *n* = 34C: *n* = 53 Personal assistants I: *n* = 31C: *n* = 22	*Pool*: Outpatients *Sample*: People using personal-assistant services and relying on a wheelchair for primary mobility. Included people with SCI, cerebral palsy/multiple sclerosis, spinal cord dysfunction, neurological dysfunction, and others (not specified)	To improve the consumer/personal-assistant relationship and increase knowledge on health and wellness issues	During life in community	6 h	*I: In-person training program:* Included information on health threat and severity of commonly occurring secondary conditions (including PUs), and preventive behaviors + training (information, role-playing) on management of personal assistance services (skills relating to listening, communication, task delegation, assertiveness, problem solving, supervisory and management role, recruitment/hiring process) *BCTs*: Instruction on how to perform the behavior, information about health consequences, behavioral practice/rehearsal, credible source *C: Received no training* *BCTs*: None coded
Norris [35], United States, Solomon 4 group design^c^ I: *n* = 78C: *n* = 49	*Pool: Inpatients* *Sample:* Patients with relatively new SCI (12–18 months post injury) to be hospitalized for at least 60 days	To encourage health behaviors through association, repetition, feedback, discussion, and rehearsal of skills and knowledge	During inpatient stay only	8 Weeks	*I: Spinal Injury Learning Sessions*: Group meetings 3 × /week to discuss: (1) introduction to spinal injury, (2) the bowel program, (3) the bladder program, and (4) the skin program. Each learning program incorporates videotaped films, illustrated learning sheets, game-type-learning activities, and reviewing of materials for group discussions *BCTs*: Problem solving, self-monitoring of behavior, instruction on how to perform the behavior, information about health consequences, salience of consequences, demonstration of the behavior, behavioral practice/rehearsal, and credible source *C: Standard in-house educational program* BCTs: None coded
Phillips [28], United States, Non-randomized, three group trial I1: *n* = 12 I2: *n* = 14C: *n* = 11	*Pool*: Outpatients *Sample*: Newly injured spinal cord individuals discharged back to community	To prevent PUs in newly injured spinal cord patients post discharge	Starting at discharge or within 2 years from discharge	10–12 Weeks	*I1: Video and phone-based telehealth:* After discharge, weekly video sessions (for 6–8 weeks) during which a nurse visually checked patient’s skin condition to monitor for PUs. Through visual contact the nurse could also help resolve problems related to wheelchairs, mattresses, and mobility about the house + weekly telephone-counseling sessions for the following 4–6 weeks (described below) *BCTs*: Problem solving, instruction on how to perform the behavior *I2: Phone-based telehealth:* Telephone-only counseling sessions to guide patients through skin checkups and assist in problem solving related to bowel, diet, or any matter of concern *BCTs*: Problem solving, instruction on how to perform the behavior *C: Standard care*: Provision of instructions on using the Shepherd Center helpline BCTs: Social support (unspecified)
Kennedy [26], United Kingdom, Non-randomized, three group trial (historical controls) I 1: *n* = 30 I 2: *n* = 11C: *n* = 9	*Pool*: Inpatients *Sample*: Patients from a SCI center	To optimize the individual’s posture, function, and tissue viability in the most appropriate seating system, and to educate patients regarding their skin care and PU prevention needs	During inpatient stay only	Unclear	*I1: Specialist seating assessment delivered before skin care needs assessment:* Posture (physical alignment) and functional ability assessed for correctability of the setup of the seating system. Included patient education and feedback (verbal and visual) provided during three assessments: (1) skin inspection using hand mirror, (2) interface pressure mapping, and (3) tissue oxygen measurement in both loaded and unloaded positions *BCTs*: Feedback on behavior, biofeedback, instruction on how to perform the behavior, information about health consequences, salience of consequences *I2: Specialist seating assessment (same as above) delivered during inpatient stay after the skin care needs assessment* *C: No specialist seating assessment (because of patients’ methicillin-resistant Staphylococcus aureus status)* BCTs: None coded

*BCT* Behavior Change Technique, *C* Control, *I* Intervention, *MS* multiple sclerosis, *NR* not reported, *PU* pressure ulcer, *SCD* spinal cord dysfunction, *SCI* spinal cord injury

^a^ This study is presented as a two-arm RCT in Garber et al. (2002; 1 control group, and intervention group) and as a three-arm RCT in Rintala et al. (2008) (two control groups and one intervention group). The papers differ in the outcomes and analyses they report, but control participants are identical across papers. Control groups in Rintala et al. (2008) are described as differing in time points for skin status assessments (quarterly vs. monthly), i.e., according to data collection methods rather than treatment received

^b^ Some education was delivered to participants in both groups prior to randomization. Although this is not described as part of treatments received per se by the authors, these BCTs were added to the list of BCTs coded to intervention and control groups as they likely influence skin care and self-management behaviors. A list of BCT definitions is available at http://www.bct-taxonomy.com/

^c^ Sample sizes for each group are reported for the intervention and control groups as a whole

The interventions tested in the 15 studies can be classified into five categories according to their primary component and mode of delivery, and irrespective of BCTs:

1. *Structured education programs* (five studies [22, 32, 34–37] including two RCTs [22, 34, 36]): Interventions consisted of educational sessions that were pre-defined in content and delivered to individuals (one study [22, 36]) or groups (four studies [32, 34, 35, 37]). Follow-up phone calls to deliver behavior reminders and motivational interviewing were included in two studies [22, 34, 36].
2. *Telehealth* (four studies [24, 25, 28, 29, 33] including three RCTs [24, 25, 29, 33]): These interventions were primarily delivered to individuals using video- or phone-based technologies. Participants were provided with health education, monitoring of skin health by study personnel, guidance on skin care behaviors, a review of their needs by a HCP, and/or advice and support to manage SCI complications including PUs (if necessary).
3. *Wheelchair skills training* (three RCTs [23, 27, 38]): These studies evaluated the same Wheelchair Skills Training program that aimed to improve participants’ performance of a range of wheelchair skills. The majority of wheelchair skills were related to mobility and ability to navigate their environment. A smaller proportion of skills related to pressure relief for skin care (“relieving weight from buttocks”).
4. *Risk assessment and feedback* (two studies [26, 31] including one RCT [31]): These interventions made the risks associated with certain body positions and behaviors salient. One study [26] provided participants with visual feedback on the effects of pressure distribution. The second study [31] consisted of providing automated (i.e., computer-generated) and in-person feedback on the risks associated with some behaviors in the prevention of secondary complications including PUs.
5. *Body-positioning skills training* (one RCT [30]): This intervention encouraged participants to change body position through demonstration, practice, reinforcement, and personalized guidance on pressure relief frequency.

The BCT taxonomy was applied to all 17 interventions (*k*) and 15 controls (*c*) group treatment descriptions. Intervention materials were used to support coding of seven [22, 23, 25, 27, 33–36, 38] (42%) of the 17 interventions reviewed (see Supplementary Information 4 for a list of used and missing intervention materials). Reasons for non-availability of intervention materials included authors no longer having copies of materials, lack of corresponding email addresses to contact authors, author preference to keep documents confidential, and author non-response.

One study [34] included the delivery of care components before randomization (i.e., instructions on how to perform skin care and biofeedback). As these were likely to influence skin care and PU outcomes, a decision was made to code them for both intervention and control group treatments. In four other studies [23, 27, 35, 38], the intervention materials used to code BCTs provided a full description of a program. It remained unclear whether the full program was implemented, but none of the authors reported adjustments or tailoring to local context.

On average, 12.3 ± 4.0 BCTs (range 8–17, median 12) were coded in the seven interventions for which intervention materials were available. In these, 6.4 ± 2.1 BCT codes (range 2–8, median 7) were unique to intervention materials (i.e., absent from intervention descriptions in published papers). For the 10 interventions for which no supporting materials were available, the average number of BCTs was 3.3 ± 1.8 BCTs (range 0–6, median 3). For control group treatments, the average number of BCTs coded was 0.8 ± 0.9 (range 0–3, median 1).

**Fig. 2:**
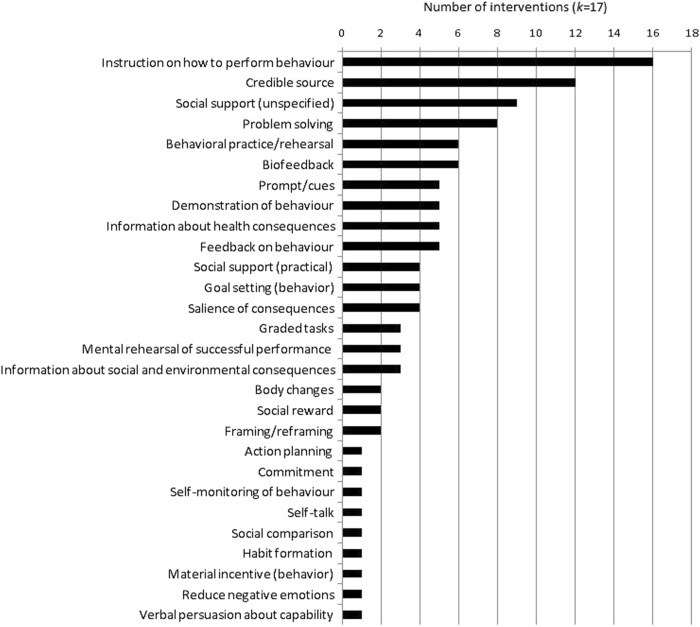
Behavior change techniques coded to the 17 interventions reviewed

The 17 interventions included 28 (30 %) of the 93 BCTs listed in the BCT taxonomy (see Fig. 2 for a graphical display, and Supplementary Information 5 for the studies they apply to). The three most commonly coded BCTs were *instructions on how to perform behavior* (*k* = 16, 94 %), *credible source* (interventions where verbal or visual communications in favor of or against the behavior are presented by a credible source, e.g., HCP; *k* = 12, 71 %), and *social support (unspecified;* *k* = 9, 53%).

Four BCTs were coded across the 15 control group treatment descriptions (see Supplementary Information 6): *social support (unspecified;* *c* = 5, 33%), *credible source* (*c* = 3, 20%), *instructions on how to perform behavior* (*c* = 2, 13%), and *biofeedback* (*c* = 2, 13%). The small number of BCTs coded reflects the low level of intervention associated with standard care, as well as the lack of description of control group treatment [39] (see Table 1).

### Aim 2: Review of intervention effectiveness (RCTs only)

Table 1 includes detailed information on key characteristics of the 10 RCTs [22–25, 27, 29–31, 33, 34, 36, 38]. Just over half of RCTs (six studies [24, 29–31, 34, 38]) were specific to people with SCI, and half (five studies [22–24, 27, 30, 36]) included a sample size of 50 participants or less. The shortest intervention length was 2 h in one RCT [31], and the longest was 2 years in two RCTs [22, 24, 36]. Only three interventions were delivered during life in the community (no association with an inpatient stay) [23, 27, 38]. Intervention groups in RCTs included 26 BCTs, the most common being identical to those listed above.

### Risk of bias

**Fig. 3:**
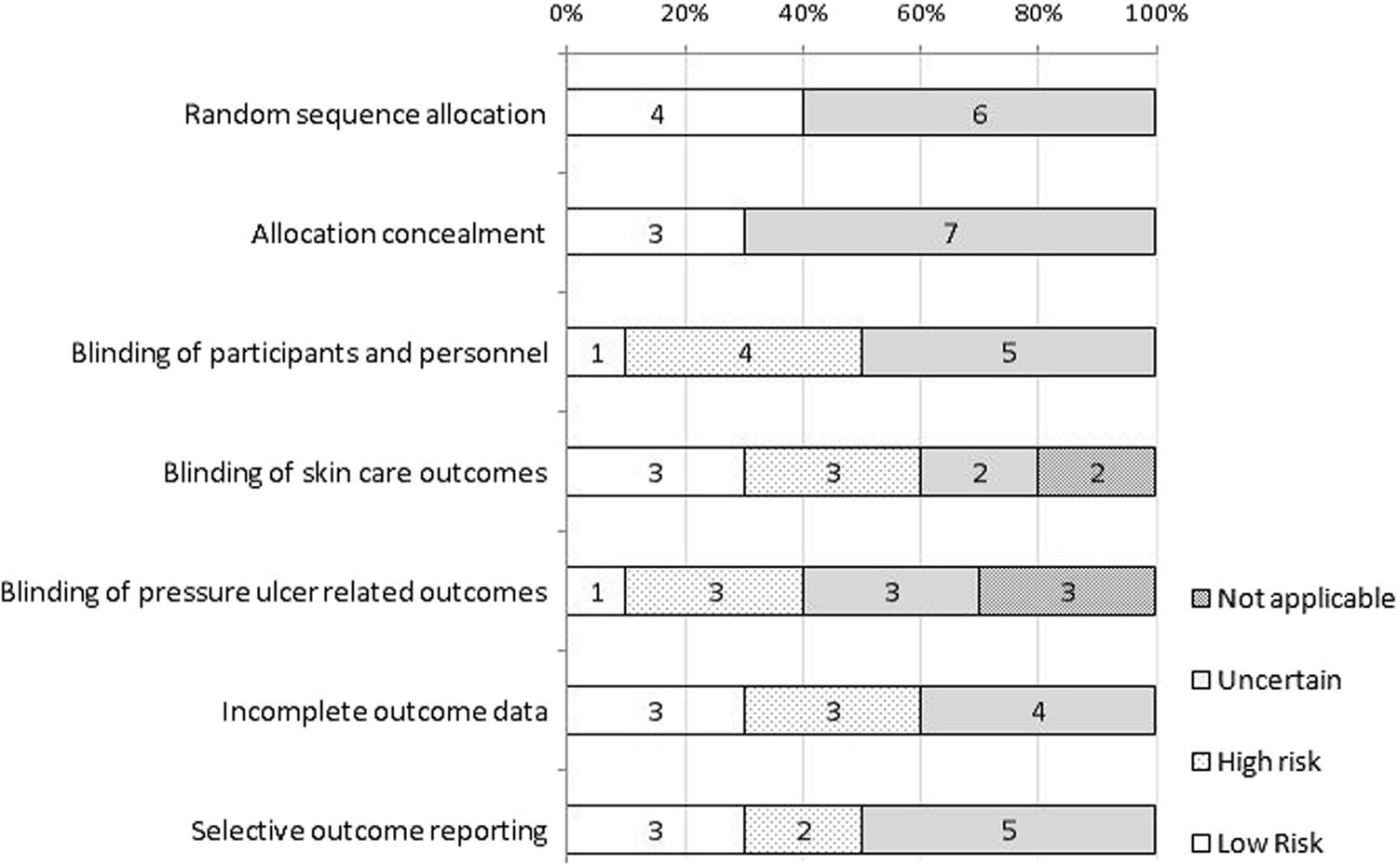
Risk of bias assessment results across the 10 randomized controlled trials reviewed. Notes: *High* high risk of bias, *low* low risk of bias, *Uncertain* unclear risk of bias. The “unclear” category was used where there was insufficient detail for a conclusion to be reached, or where there was no published/registered study protocol available (item on selective outcome reporting). A decision about the selective outcome reporting item was reached by comparing outcomes reported on to measures outlined in registered or published protocols. If a protocol was not available, this item was marked as “unclear”. In addition, items on blinding of outcomes were rated “not applicable” if skin care outcomes or pressure-ulcer-related outcomes were not measured

Figure 3 is a graphical display of overall risk of bias in the 10 RCTs [22–25, 27, 29–31, 33, 34, 36, 38]. Risk of bias results for each study can be found in Supplementary Information 7. Only three studies were judged not to have a high risk of bias in any of the seven domains evaluated [29–31]. Blinding of participants and personnel, and incomplete outcome data reporting were the two risk of bias domains most frequently rated “high risk”. A large proportion of the risk assessment domains could not be rated because of incomplete or unclear information (“unclear”). Allocation concealment was the domain with the greatest number of “unclear” ratings.

### Intervention effects on outcomes

A narrative summary of findings is presented below by outcome measure, with Supplementary Information 8 detailing study findings. Effect sizes could be calculated in four studies. They are included below.

### Mediators of skin care behaviors

Mediators of skin care measured included knowledge, self-efficacy, or skills relating to skin care.

#### Knowledge about skin care and PU prevention

A self-report measure of knowledge was included in three studies [22, 31, 34, 36]. Two [22, 34, 36] studies used the same Pressure Ulcer Knowledge test (unclear psychometric properties). Complete reporting of knowledge data was available in one study [22, 36] that found that an enhanced educational inpatient program including 4 h of individualized learning sessions resulted in significantly greater knowledge about PUs compared to a short education program delivered over 1–2 h. The effect size (Hedges’ *g*) was 0.71 (95% CI 0.06, 1.36).

#### Self-efficacy relating to skin care

Self-efficacy for skin care (i.e., confidence in one’s ability to prevent and manage PUs) was measured in one study [34] using a validated scale adapted for PUs. No data were reported or provided by authors.

#### Skills relating to skin care

Four studies assessed skills relating to skin care [23, 27, 30, 38]. Three studies [23, 27, 38] testing a wheelchair skills training program included a trainer administering a validated Wheelchair Skills Test Questionnaire, which involves the assessment of over 30 individual wheelchair skills including one item on pressure relief (ability to “relieve pressure from buttocks”). Individual scores for the pressure relief skill were not reported in any of these studies. Although significant improvements in overall wheelchair skill summary scores were observed for the intervention group in all three studies [23, 27, 38], none of the participants worked on the pressure relief skill. This was by choice in two studies [23, 38], or the result of successfully completing this skill at baseline in the third study [27] (Ozturk A., personal communication, 14 February 2017).

A fourth study [30] that measured skills tested a 4-week inpatient body-positioning training program in which patients were taught how to change body position, and received social reinforcement and encouragements to follow an individualized body-positioning schedule. An HCP assessed the degree of assistance required for body change positions, using a self-developed scale with face validity. A larger decrease in assistance needed was observed in intervention participants at 4 weeks compared to controls. The sample size, however, was small (*n* = 10).

### Skin care behaviors

Four studies included a behavioral measure of skin care [25, 30, 31, 33, 34]; however, data were only available for three studies [25, 30, 33, 34]. Behavioral measures included items relating to skin checks, pressure relief (e.g., frequency of body position changes and intervals of prolonged skin pressure), wheelchair cushion checks, alcohol and substance abuse, help seeking with new or worsening skin breakdown, and keeping skin clean and dry. The two studies [30, 34] for which between-group differences were tested statistically yielded mixed findings. The first study [34] compared a self-management and skills training (problem solving, self-monitoring, and mood and stress management) intervention plus motivational interviewing follow-up phone calls to a standard care self-management intervention without skill training or motivational interviewing. The proportion of skin care behaviors performed (measured using a self-developed Skin Care Behavior Checklist with unclear psychometric properties) did not significantly increase at 3 and 6 month follow-ups in the intervention group compared to controls, and the effect size CIs crossed zero (Hedges’ *g* = 0.13, 95% CI −0.19, 0.46). In contrast, results of the second study [30] (*n* = 10) indicated that a 4-week inpatient body-positioning training program increased the frequency of pressure relief behaviors measured via HCP observations. Finally, descriptive statistics performed on the raw data sent to our research team for a third study [25, 33] evaluating a telehealth intervention (Houlihan B., personal communication, 3 April 2017) suggested that the greate st group differences at follow-up were on two pressure relief items (sitting and in bed), with the intervention group performing pressure relief more frequently. The effect size calculated using the total scale score indicated a medium effect and CIs did not cross zero (Hedges’ *g* = 0.60, 95% CI 0.25, 0.95).

### Clinical outcomes related to pressure ulcers

Seven [22, 24, 25, 29–31, 33, 34, 36] of the ten RCTs measured skin status. Measurement methods varied (participant self-report [22, 29, 36], physical examinations carried out by HCPs [24, 25, 30, 33], HCP assessment of photos taken by patients/carer or patient self-report, and combined with verification of medical records [31, 34]). Participants’ baseline skin status varied across studies (samples with and without PUs [25, 30, 31, 33], with both closed and open wounds [34], with a PU that had healed after surgical repair [22, 36], or PU-free [24]).

With the exception of one study [29], all studies reported some follow-up data on this outcome.

Only one intervention [22, 36] significantly improved skin status compared to controls. These results suggest that up to 4 h of structured education was more effective than the standard 1–2 h education program in preventing PU recurrence after surgical repair [22, 36] (OR = 0.17, 95% CI 0.04, 0.68). Three studies [25, 31, 33, 34] evaluating telehealth, self-management sessions plus motivational interviewing, and a risk assessment and feedback on PU risk factors' intervention did not find skin status variables (skin worsening, time to skin worsening, and PU development) to be significantly influenced (where calculated, OR 95% CIs crossed 1). A significant subgroup analysis difference in PU development was noticed in the telehealth trial [25, 33] with intervention group females developing considerably fewer PUs than males at 6 months. The two last trials assessing PU prevalence and/or severity did not conduct statistical tests [24, 30]. One [24] of these was a telehealth trial for which the OR 95% CIs crossed 1, the other did not report the data clearly [30].

Only one study [34] measured health-care utilization for skin problems and number of days on bedrest. Results were reported incompletely, but health-care utilization was not significantly influenced by the intervention.

## Discussion

To the best of our knowledge, this is the first study to examine the content of SCI skin care interventions using the BCT taxonomy. Twenty-eight of ninety-three defined BCTs were coded in 17 interventions. This is a slightly higher number of BCTs compared to those identified in a review of home-based rehabilitation interventions [40] that includes roughly the same number of studies, and a slightly smaller number than a review of interventions for chronic back pain and arthritis that includes 25 papers [41]. The number of BCTs included in interventions is likely to vary according to patients’ health behaviors, outcomes targeted, and the complexity of the medical condition. SCI is recognized to be very complex with several secondary complications to self-manage, which could explain the higher number of BCTs identified compared to other studies. The number of BCTs included may also depend on researchers’ and clinicians’ perceptions of what is required for behavior change. Some may still believe that behavior change can be achieved with simple knowledge-based interventions. Evidence suggests that increasing knowledge is necessary, but not sufficient, for behavior change [42]. PU prevention requires interventions that encourage routine performance of multiple skin care behaviors, develop patients’ multidisciplinary knowledge, and include personalized intervention components. Repeated exposure to multiple BCTs delivered in different formats is likely required for behavior change and maintenance.

From a methodological perspective, the number of BCTs identified is related to the quality of intervention descriptions. The current study underlines the value of searching or contacting authors for intervention materials, as the number of BCTs coded using intervention materials was four times that of interventions for which none were available. The poor description of interventions in published materials underlines the need to report intervention content using published guidelines (e.g., Template for Intervention Description and Replication checklist [43]).

Sixty-five BCTs from the BCT taxonomy were not coded to any intervention, suggesting that many techniques from behavioral science remain unexplored despite their potential impact on skin care in SCI. Similar to other reviews on self-management programs [40, 41], the most commonly identified BCTs included instructions on how to perform behavior, credible source, and social support (unspecified). Testing other BCTs can advance the science of self-management in SCI and our understanding of the underlying mechanisms through which they exert their effect. Not all BCTs in the BCT taxonomy may be applicable, feasible, or appealing in the SCI context (e.g., future punishment, pharmacological support, and paradoxical instructions). SCI researchers may benefit from conducting research to identify factors that influence skin care in SCI. These findings considered alongside a matrix of behavior change techniques linked to theoretical predictors of behavior [44] can help SCI researchers and clinicians select BCTs to include in skin care interventions. Factorial designs can be used to test multiple BCTs and can provide information on their individual and interaction effects.

The second aim of this study was to focus on RCTs to examine the effects of skin care self-management interventions on skin care and PU-related outcomes. Heterogeneous patient populations make RCTs difficult in rehabilitation research and the identification of 10 RCTs in this review is encouraging. Poor reporting, small sample sizes, variation in outcome measures and their psychometric properties, and mixed findings make it challenging to reach conclusions about the effects of the interventions reviewed. Effect sizes for the few studies and outcomes for which they could be calculated showed promise, particularly as their calculation was based on the longest follow-up when attrition rates tend to be highest. Reliance on shorter follow-up data would likely not influence findings as they were only available in one study [34]. Another finding from our review on intervention effectiveness suggests that wheelchair skills training programs that rely on patients selecting skills to work on are not ideal platforms through which to influence skin care, as patients prioritize everyday mobility skills. Making them mandatory to work on or a core component of these interventions is advised.

Skin status was the most commonly measured outcome yet comparisons across studies are difficult because of variation in measurement methods, length of follow-up, and baseline characteristics. Despite randomization, differences in skin status at baseline in particular can make results difficult to interpret if insufficient data are reported at follow-up on whether the PUs observed are new or the same as at baseline, their location, and their staging. Accurate and reliable PU measurements are recognized to be difficult [45]. It is recommended that researchers be precise in reporting data for this outcome, including detailed documentation of observed PU stages and anatomical locations [45]. This will enable reliable local and international benchmarking between health-care settings and facilities. The strong need for consistency in design and reporting was underlined by an International Guideline Recommendation Group [45], and it should be noted that there are internationally accepted standards available for reporting information about PUs in SCI [46]. In terms of study design, the majority of RCTs in this review included short-term follow-ups (6 months or less). Longer follow-ups are more likely to allow for behavioral and clinical changes. In addition, very few of the identified RCTs included more than one follow-up. Repeated assessments will help identify short- and long-term changes, and will allow for statistical analyses (e.g., mediation analyses) that investigate pathways of change.

Results of a posteriori analyses in one [25, 33] of the RCTs reviewed suggested that females benefitted from the intervention more than males. This may be related to male gender being a risk factor in PU development [47], or possibly to females responding better to self-management interventions [48]. Future work may benefit from planning such analyses during study design to ensure that gender interaction effects can be studied with sufficient statistical power.

Our risk of bias-assessment results suggests that much of the information required in papers to reach a judgment was unavailable (“uncertain” risk of bias assessment, Fig. 3). Researchers should use the Cochrane risk of bias tool to guide reporting on internal validity. Blinding of participants and personnel, and incomplete outcome data reporting were the two risk of bias domains most frequently rated “high risk”. A discussion of the challenges to blind participants and personnel in behavioral interventions proposes some procedures to help reduce risk of bias [49]. Reporting of outcome data should not be influenced by the direction of the findings and incomplete reporting of outcome data should be justified.

Only three of the ten RCTs reviewed evaluated interventions delivered to community-dwelling people with SCI. High rates of PUs after discharge suggest that community-based approaches are needed in PU prevention efforts. Networks of community care organizations and national registries can be used to reach people with a SCI in the community, and web-based technologies can be used to facilitate intervention delivery and receipt. In a Canadian survey [50], people with SCI confirmed the importance of developing community-based self-management programs, indicating that the Internet was the most appropriate delivery mode. In a separate survey on their information needs [51], medical issues relating to SCI (e.g., skin, bladder, bowel, and pain) ranked first. People with a new SCI diagnosis may be more receptive to preventive interventions after adjusting to life with SCI.

A limitation of this work is that non-randomized studies with one research group were not included in our review of intervention content. More inclusive inclusion criteria may have led to the identification of additional BCTs. In light of this, the consistency of our results with the number of BCTs identified in the number of BCTs identified in other reviews of self-management interventions is reassuring. Another limitation is that study characteristics made it difficult to conduct a meta-analysis or meta-regression to identify effective BCTs. In addition, descriptions of intervention components often did not specify the health behaviors they targeted (including skin care). Other reviews on physical activity and healthy eating [52] have included such analyses, likely because of the greater number of eligible studies, better reporting of intervention protocols and effects, and consistency in outcomes.

Strengths of this work include the robust search strategy and attempts to contact authors for intervention materials and outcome data. Our use of a standardized taxonomy of BCTs also contributes to building a science of self-management in SCI by encouraging cross-study comparisons and common terminology in future studies.

This systematic review has identified the most commonly used BCTs in SCI skin care self-management interventions, as well as the potential promise of some of the tested SCI skin care interventions. Future work in this area would benefit from larger-scale studies, consistent use of validated outcome measures, and testing of a greater variety of BCTs.

## Electronic supplementary material

Search strategies

Behavior change technique taxonomy

List of excluded references at full-text

Used and missing intervention materials

Behavior change techniques coded in intervention groups

Behavior change techniques coded in control groups

Risk assessment results for the 10 randomized trials reviewed

Summary of effectiveness findings for the 10 randomized controlled trials reviewed
