# Supplementary material for: Self-management interventions for skin care in people with a spinal cord injury: part 1—a systematic review of intervention content and effectiveness
Source: Spinal Cord. 2018 May 25;56(9):823–36. doi: 10.1038/s41393-018-0138-3 (PMC6128818; doi:10.1038/s41393-018-0138-3)
Supplement: Supplementary file 1 — Search strategies [file 41393_2018_138_MOESM1_ESM.docx]

**Supplementary File 1.** Search Strategies

Electronic bibliographic database searches were run on 23^rd^ February 2016.

- 1. **Electronic bibliographic databases**

MEDLINE

1.exp spinal cord injuries/

2.spinal cord/

3.cervical cord/

4.exp Spinal Cord Ischemia/

5. exp spinal injuries/

6. exp spinal cord diseases/

7. exp myelitis/

8.paralysis/

9.exp paraplegia/

10. quadriplegia/

11. (central cord syndrome).ti,ab

12. (spinal cord) adj1 (dysfunction? or injur* or disease? or syndrome or trauma or lesion? or lacerat* or transaction? or fracture? or contusion or ischemia).ti,ab

13. (spine or spinal) adj1 (disease? or injur* or fracture?).ti,ab

14. (paralysis).ti,ab

15. (quadr?plegi*).ti,ab

16. (tetraplegi*).ti,ab

17. (paraplegi*).ti,ab

18. (Brown-Sequard).ti,ab

19. (myelopathy).ti,ab

20. (myelitis).ti,ab

21. (SCI).ti,ab

22. or/1-21

23. exp skin ulcer/

24. exp pressure ulcer/

25. ulcer/

26. (bedsore* or bed sore*).ti,ab

27. (skin) adj1 (ulcer* or care or integrity or management or breakdown or wound? or lesion? or promot*).ti,ab

28. (pressure) adj1 (ulcer* or injur* or damag* or sore* or wound*).ti,ab

29. (decubitus or decubital).ti,ab

30. (secondary) adj2 (complication* or condition*).ti,ab

31. (medical complication*).ti,ab

32. or/23-31

33. disease management/

34. primary prevention/

35. secondary prevention/

36. tertiary prevention/

37. preventive health services/

38. Early Medical Intervention/

39. Health promotion/

40. self-administration/

41. self-medication/

42. self-care/

43. risk reduction behavior/

44. exp consumer participation/

45. health behavior/

46. exp patient compliance/

47. self-examination/

48. exp patient-centered care/

49. activities of daily living/

50. exp life style/

51. (self) adj1 (help or manage* or care or determination or administr* or medicat* or treat*).ti,ab

52. (disease management).ti,ab

53. (expert patient*).ab, ti

54. (empower*).ti,ab

55. (promot*) adj4 (health or behavio?r or well-being)

56. (prevent*).ti,ab

57. (participation) adj1 (patient* or consumer*).ti,ab

58. (support).ti,ab

59. (behavio?r*) adj1 (treatment* or therap* or health or modif* or chang* or intervention).ti,ab

60. program evaluation/

61. (program? or programme?).ti,ab

62. (workshop).ti,ab

63. behavior therapy/

64. (training).ti,ab

65. (skill*).ti,ab

66. (change) adj2 (strategies or strategy).ti,ab

67. (patient-cent?red care).ti,ab

68.(patient-focus*).ti,ab

69.(adjustment).ti,ab

70. (activit* of daily living).ti,ab

71. ((life style or lifestyle) adj5 (modif* or chang* or alter* or rehab* or intervention* or management)).ti,ab

72.(education).ti,ab

73. Health education/

74. Consumer Health information/

75. Patient education as topic/

76. (rehabilitation).ti,ab

77. Telerehabilitation/

78.(telemedicine or telehealth or telecare or telerehabilitation).ti,ab

79. (complian*).ti,ab

80. (adheren*).ti,ab

81. or/33-80

82. 32 AND 81

83. exp skin care/

84. or/82-83

85. 22 AND 84

EMBASE

1. exp spinal cord injury/

2. exp spine injury/

3. spinal paralysis/

4. spinal cord/

5. spinal cord ischemia/

6. paraplegia/

7. quadriplegia/

8. exp spinal cord disease/

9. exp myelitis/

10. (central cord syndrome).ti,ab

11. (spinal cord) adj1 (dysfunction? or injur* or disease? or syndrome or trauma or lesion? or lacerat* or transaction? or fracture? or contusion or ischemia).ti,ab

12. (spine or spinal) adj1 (disease? or injur* or fracture?).ti,ab

13. (paralysis).ti,ab

14. (quadr?plegi*).ti,ab

15. (tetraplegi*).ti,ab

16. (paraplegi*).ti,ab

17. (Brown-Sequard).ti,ab

18. (myelopathy).ti,ab

19. (myelitis).ti,ab

20. (SCI).ti,ab

21. or/1-20

22. exp skin ulcer/

23. ulcer/

24. (bedsore* or bed sore*).ti,ab

25. (skin) adj1 (ulcer* or care or integrity or management or breakdown or wound? or lesion? or promot*).ti,ab

26. (pressure) adj1 (ulcer* or injur* or damag* or sore* or wound*).ti,ab

27. (decubitus or decubital).ti,ab

28. (secondary) adj2 (complication* or condition*).ti,ab

29. (medical complication*).ti,ab

30. or/22-29

31. disease management/

32. exp self-care/

33. primary prevention/

34. secondary prevention/

35. preventive medicine/

36. preventive health services/

37. early intervention/

38. health promotion/

39. health education/

40. patient education/

41. health behavior/

42. risk reduction/

43. patient participation/

44. exp patient compliance/

45. self-examination/

46. daily life activity/

47. lifestyle modification/

48. drug self-administration/

49**.**  behavior modification/

50**.**  behavior change/

51. prevention study/

52. (self) adj1 (help or manage* or care or determination or administr* or medicat* or treat*).ti,ab

53. (disease management).ti,ab

54. (expert patient*).ab, ti

55. (empower*).ti,ab

56. (promot*) adj4 (health or behavio?r or well-being)

57. (prevent*).ti,ab

58. (participation) adj1 (patient* or consumer*).ti,ab

59. (support).ti,ab

60. (behavio?r*) adj1 (treatment* or therap* or health or modif* or chang* or intervention).ti,ab

61. program evaluation/

62. (program? or programme?).ti,ab

63. (workshop).ti,ab

64. (training).ti,ab

65. (skill*).ti,ab

66. (change) adj2 (strategies or strategy).ti,ab

67. (patient-cent?red care).ti,ab

68. (patient-focus*).ti,ab

69. (adjustment).ti,ab

70. (activit* of daily living).ti,ab

71. ((life style or lifestyle) adj5 (modif* or chang* or alter* or rehab* or intervention* or management)).ti,ab

72.(education).ti,ab

73. (rehabilitation).ti,ab

74.(telemedicine or telehealth or telecare or telerehabilitation).ti,ab

75. (complian*).ti,ab

76. (adheren*).ti,ab

77. or/31-76

78. 30 AND 77

79. skin care/

80. or/78-79

81. 21 AND 80

PSYCINFO

1. exp spinal cord injuries/

2. spinal cord/

3. paralysis/

4. paraplegia/

5. quadriplegia/

6. exp myelitis/

7. (central cord syndrome).ti,ab

8. (spinal cord) adj1 (dysfunction? or injur* or disease? or syndrome or trauma or lesion? or lacerat* or transaction? or fracture? or contusion or ischemia).ti,ab

9. (spine or spinal) adj1 (disease? or injur* or fracture?).ti,ab

10. (paralysis).ti,ab

11. (quadr?plegi*).ti,ab

12. (tetraplegi*).ti,ab

13 (paraplegi*).ti,ab

14. (Brown-Sequard).ti,ab

15. (myelopathy).ti,ab

16. (myelitis).ti,ab

17. (SCI).ti,ab

18. or/1-17

19. skin disorders/

20. (bedsore* or bed sore*).ti,ab

21. (skin) adj1 (ulcer* or care or integrity or management or breakdown or wound? or lesion? or promot*).ti,ab

22. (pressure) adj1 (ulcer* or injur* or damag* or sore* or wound*).ti,ab

23. (decubitus or decubital).ti,ab

24. (secondary) adj2 (complication* or condition*).ti,ab

25. (medical complication*).ti,ab

26. or/19-25

27. disease management/

28. client education/

29. health knowledge/

30. treatment compliance/

31. health promotion/

32. prevention/

33. preventive medicine/

34. self-medication/

35. drug self-administration/

36. self-care skills/

37. client participation/

38. health behavior/

39. illness behavior/

40. self- help techniques/

41. self-management/

42. treatment compliance/

43. "self examination (medical)"/

44. activities of daily living/

45. lifestyle changes/

46. program evaluation/

47. behavior modification/

48. exp behavior change/

49. (self) adj1 (help or manage* or care or determination or administr* or medicat* or treat*).ti,ab

50. (disease management).ti,ab

51. (expert patient*).ab, ti

52. (empower*).ti,ab

53. (promot*) adj4 (health or behavio?r or well-being)

54 (prevent*).ti,ab

55. (participation) adj1 (patient* or consumer*).ti,ab

56. (support).ti,ab

57. (behavio?r*) adj1 (treatment* or therap* or health or modif* or chang* or intervention).ti,ab

58. (program? or programme?).ti,ab

59. (workshop).ti,ab

60. (training).ti,ab

61. (skill*).ti,ab

62. (change) adj2 (strategies or strategy).ti,ab

63. (patient-cent?red care).ti,ab

64.(patient-focus*).ti,ab

65.(adjustment).ti,ab

66. (activit* of daily living).ti,ab

67. ((life style or lifestyle) adj5 (modif* or chang* or alter* or rehab* or intervention* or management)).ti,ab

68.(education).ti,ab

69. (rehabilitation).ti,ab

70.( telemedicine or telehealth or telecare or telerehabilitation).ti,ab

71. (complian*).ti,ab

72. (adheren*).ti,ab

73. or/27-72

74. 18 AND 26 AND 73

CINAHL

S1. MH "spinal cord injuries+"

S2. MH "Spinal Cord"

S3. MH "Cervical Cord"

S4. MH "Spinal Injuries+"

S5. MH Spinal cord diseases+"

S6. MH "Myelitis+"

S7. MH "Paralysis"

S8. MH "paraplegia+"

S9. MH "Quadriplegia"

S10. MH "spinal diseases+"

S11. TI (central cord syndrome) or AB (central cord syndrome)

S12. TI (spinal cord) N1 (dysfunction? or injur* or disease? or syndrome or trauma or lesion? or lacerat* or transaction? or fracture? or contusion or ischemia) or AB (spinal cord) N1 (dysfunction? or injur* or disease? or syndrome or trauma or lesion? or lacerat* or transaction? or fracture? or contusion or ischemia)

S13. TI (spine or spinal) N1 (disease? or injur* or fracture?) or AB (spine or spinal) N1 (disease? or injur* or fracture?)

S14. TI (paralysis) or AB (paralysis)

S15. TI (quadr?plegi*) or AB (quadr?plegi*)

S16. TI (tetraplegi*) or AB (tetraplegi*)

S17. TI (paraplegi*) or AB (paraplegi*)

S18. TI (brown-sequard) or AB (brown-sequard)

S19. TI (myelopathy) or AB (myelopathy)

S20. TI (myelitis) or AB (myelitis)

S21. TI (SCI) or AB (SCI)

S22. or/S1-S21

S23. MH "Skin Ulcer"

S24. MH "pressure ulcer+"

S25.TI (bedsore? or bed sore?) or AB ((bedsore? or bed sore?)

S26. TI (skin) N1 (ulcer* or care or integrity or management or breakdown or wound? or lesion? or promot*) or AB (skin) N1 (ulcer* or care or integrity or management or breakdown or wound? or lesion? or promot*)

S27. TI (pressure) N1 (ulcer* or injur* or damag* or sore? or wound?) or AB (pressure) N1 (ulcer* or injur* or damag* or sore? or wound?)

S28. TI (decubitus or decubital) or AB (decubitus or decubital)

S29. TI (secondary) N2 (complication? or condition?) or AB (secondary) N2 (complication? or condition?)

S30. TI (medical complication?) or AB (medical complication?)

S31. or/S23-S30

S32. MH "Disease Management"

S33. MH "Preventive Health Care"

S34. MH "Primary Health Care"

S35. MH "Secondary Health Care"

S36. MH "Tertiary Health Care"

S37. MH "Early intervention"

S38. MH "health promotion"

S39. MH "self administration"

S40. MH "self medication"

S41. MH "self care"

S42. MH "self care agency"

S43. MH "consumer participation"

S44. MH "consumer health information"

S45. MH "behavioral changes"

S46. MH "health behavior"

S47. MH "patient compliance+"

S48. MH "Patient Centered Care"

S49. MH "activities of daily living"

S50. MH "Life Style Changes"

S51. MH "Program evaluation"

S52. MH "Health education"

S53. TI (self) N1 (help or manage* or care or determination or administr* or medicat* or treat*) or AB (self) N1 (help or manage* or care or determination or administr* or medicat* or treat*)

S54. TI (disease management) or AB (disease management)

S55. TI (expert patient*) or AB (expert patient*)

S56. TI (empower*) or AB (empower*)

S57. TI (promot*) adj4 (health or behavio?r or well-being) or AB (promot*) adj4 (health or behavio?r or well-being)

S58. TI (prevent*) or AB (prevent*)

S59. TI (participation) N1 (patient* or consumer*) or AB (participation) N1 (patient* or consumer*)

S60. TI (support) or AB (support)

S61. TI (behavio?r*) N1 (treatment* or therap* or health or modif* or chang* or intervention) or AB (behavio?r*) N1 (treatment* or therap* or health or modif* or chang* or intervention)

S62. TI (program? or programme?) or AB (program? or programme?)

S63. TI (workshop) or AB (workshop)

S64. TI (training) or AB (training)

S65. TI (skill*) or AB (skill*)

S66. TI (change) N2 (strategies or strategy) or AB (change) N2 (strategies or strategy)

S67. TI (patient-cent?red care) or AB (patient-cent?red care)

S68. TI (patient-focus*) or AB (patient-focus*)

S69. TI (adjustment) or AB (adjustment)

S70. TI (activit* of daily living) or AB (activit* of daily living)

S71. TI ((life style or lifestyle) N5 (modif* or chang* or alter* or rehab* or intervention* or management)) or AB ((life style or lifestyle) N5 (modif* or chang* or alter* or rehab* or intervention* or management))

S72. TI (education) or AB (education)

S73. TI (rehabilitation) or AB (rehabilitation)

S74 TI (telemedicine or telehealth or telecare or telerehabilitation) or AB (telemedicine or telehealth or telecare or telerehabilitation)

S75. TI (complian*) or AB (complian*)

S76. TI (adheren*) or (adheren*)

S77. or/S32-S76

S78. S31 AND S77

S79. MH "skin care"

S80. or/S78-79

S81. S22 AND S80

CENTRAL

1. (central cord syndrome).ti,ab

2. (spinal cord) adj1 (dysfunction? or injur* or disease? or syndrome or trauma or lesion? or lacerat* or transaction? or fracture? or contusion or ischemia).ti,ab

3. (spine or spinal) adj1 (disease? or injur* or fracture?).ti,ab

4. (paralysis).ti,ab

5. (quadr?plegi*).ti,ab

6. (tetraplegi*).ti,ab

7. (paraplegi*).ti,ab

8. (Brown-Sequard).ti,ab

9. (myelopathy).ti,ab

10. (myelitis).ti,ab

11. (SCI).ti,ab

12. or/1-11

13. (bedsore* or bed sore*).ti,ab

14. (skin) adj1 (ulcer* or care or integrity or management or breakdown or wound? or lesion? or promot*).ti,ab

15. (pressure) adj1 (ulcer* or injur* or damag* or sore* or wound*).ti,ab

16. (decubitus or decubital).ti,ab

17. (secondary) adj2 (complication* or condition*).ti,ab

18. (medical complication*).ti,ab

19. or/13-18

20. (self) adj1 (help or manage* or care or determination or administr* or medicat* or treat*).ti,ab

21. (disease management).ti,ab

22. (expert patient*).ab, ti

23. (empower*).ti,ab

24. (promot*) adj4 (health or behavio?r or well-being)

25. (prevent*).ti,ab

26. (participation) adj1 (patient* or consumer*).ti,ab

27. (support).ti,ab

28. (behavio?r*) adj1 (treatment* or therap* or health or modif* or chang* or intervention).ti,ab

29. (program? or programme?).ti,ab

30. (workshop).ti,ab

31. (training).ti,ab

32. (skill*).ti,ab

33. (change) adj2 (strategies or strategy).ti,ab

34. (patient-cent?red care).ti,ab

35. (patient-focus*).ti,ab

36. (adjustment).ti,ab

37. (activit* of daily living).ti,ab

38. ((life style or lifestyle) adj5 (modif* or chang* or alter* or rehab* or intervention* or management)).ti,ab

39.(education).ti,ab

40. (rehabilitation).ti,ab

41.( telemedicine or telehealth or telecare or telerehabilitation).ti,ab

42. (complian*).ti,ab

43. (adheren*).ti,ab

44. or/20-43

45. 12 AND 19 AND 44

PEDRO

1. spin* AND self-manag*

2. spin* AND ulcer*

3. spin* AND self-care

4. spin* AND behavio*

5. spin* AND skin

6. spin* AND pressure AND prevent*

Note: These searches were limited to title and abstract (by default)

ERIC

1. (spine or spinal) AND (self-manag* or ulcer* or behavio* or skin).ti,ab

2. (spine or spinal) AND (pressure) AND (prevent*).ti,ab

Note: the above terms were searched across: **title, author, source, abstract** and **descriptor (by default)**

CIRRIE

1. spinal cord injuries (subject) AND self-manage (title)

2. spinal cord injuries (subject) AND self care (subject)

3. spinal cord injuries (subject) AND ulcer (title)

4. spinal cord injuries (subject) AND pressure sores (subject)

5. spinal cord injuries (subject) AND behavior modification (subject)

6. spinal cord injuries (subject) AND skin (title)

REHABDATA

These combinations were searched using the 'specific field' search feature limited to abstracts

1. ab(spinal cord injur*) AND ab(self-manag*)

2. ab(spinal cord injur*) AND ab(behavio*) AND ab(ulcer*)

3. ab(spinal cord injur*) AND ab(ulcer*) AND ab(prevent*)

4. ab(spinal cord injur*) AND ab(self-care)

5. ab(spinal cord injur*) AND ab(skin)

6. ab(spinal cord injur*) AND ab(pressure) AND ab(prevent*)

- 1. **Clinical trial registry platforms**

Clinical trial registry platforms were searched on 21^st^ June 2016.

World Health Organization International Clinical Trials Registry

Keywords used: spinal cord injury or SCI

Meta-Register of Controlled Trials

Keywords used in two independent searches:

1. spinal cord injury
2. (pressure AND ulcer) OR (pressure AND sore) OR (bed AND sore) OR decubitus
