# Supplementary material for: Self-management interventions for skin care in people with a spinal cord injury: part 1—a systematic review of intervention content and effectiveness
Source: Spinal Cord. 2018 May 25;56(9):823–36. doi: 10.1038/s41393-018-0138-3 (PMC6128818; doi:10.1038/s41393-018-0138-3)
Supplement: Supplementary file 2 — Behavior change technique taxonomy [file 41393_2018_138_MOESM2_ESM.docx]

**Supplementary File 2**. Behaviour change technique taxonomy

| **1.Goals and planning**  1.1. Goal setting (behavior)  1.2. Problem solving  1.3. Goal setting (outcome)  1.4. Action planning  1.5. Review behavior goal(s)  1.6. Discrepancy between current behavior and goal  1.7. Review outcome goal(s)  1.8. Behavioral contract  1.9. Commitment  **2.Feedback and monitoring**  2.1 Monitoring of behavior by others without feedback  2.2. Feedback on behaviour  2.3. Self-monitoring of behaviour  2.4. Self-monitoring of outcome(s) of behaviour  2.5. Monitoring of outcome(s) of behavior without feedback  2.6. Biofeedback  2.7. Feedback on outcome(s) of behavior  **3.Social support**  3.1. Social support (unspecified)  3.2. Social support (practical)  3.3. Social support (emotional)  **4.Shaping knowledge**  4.1 Instruction on how to perform the behavior  4.2. Information about Antecedents  4.3. Re-attribution  4.4. Behavioral experiments  **5.Natural consequences**  5.1. Information about health consequences  5.2. Salience of consequences  5.3. Information about social and environmental consequences  5.4. Monitoring of emotional consequences  5.5. Anticipated regret  5.6. Information about emotional consequences  **6.Comparison of behaviour**  6.1. Demonstration of the behavior  6.2. Social comparison  6.3. Information about others’ approval  **7.Associations**  7.1. Prompts/cues  7.2. Cue signalling reward  7.3. Reduce prompts/cues  7.4. Remove access to the reward  7.5. Remove aversive stimulus  7.6. Satiation  7.7. Exposure  7.8. Associative learning  **8.Repetition and substitution**  8.1. Behavioral practice/rehearsal  8.2. Behavior substitution  8.3. Habit formation  8.4. Habit reversal  8.5. Overcorrection  8.6. Generalisation of target behavior  8.7. Graded tasks | **9.Comparison of outcomes**  9.1. Credible source  9.2. Pros and cons  9.3. Comparative imagining of future outcomes  **10.Reward and threat**  10.1. Material incentive (behavior)  10.2. Material reward (behavior)  10.3. Non-specific reward  10.4. Social reward  10.5. Social incentive  10.6. Non-specific incentive  10.7. Self-incentive  10.8. Incentive (outcome)  10.9. Self-reward  10.10. Reward (outcome)  10.11. Future punishment  **11.Regulation**  11.1. Pharmacological support  11.2. Reduce negative emotions  11.3. Conserving mental resources  11.4. Paradoxical instructions  **12.Antecedents**  12.1. Restructuring the physical environment  12.2. Restructuring the social environment  12.3. Avoidance/reducing exposure to cues for the behavior  12.4. Distraction  12.5. Adding objects to the environment  12.6. Body changes  **13.Identity**  13.1. Identification of self as role model  13.2. Framing/reframing  13.3. Incompatible beliefs  13.4. Valued self-identify  13.5. Identity associated with changed behavior  **14.Scheduled consequences**  14.1. Behavior cost  14.2. Punishment  14.3. Remove reward  14.4. Reward approximation  14.5. Rewarding completion  14.6. Situation-specific reward  14.7. Reward incompatible behavior  14.8. Reward alternative behavior  14.9. Reduce reward frequency  14.10. Remove punishment  **15.Self-belief**  15.1. Verbal persuasion about capability  15.2. Mental rehearsal of successful performance  15.3. Focus on past success  15.4. Self-talk  **16.Covert learning**  16.1. Imaginary punishment  16.2. Imaginary reward  16.3. Vicarious consequences |
| --- | --- |

Note: List of BCT techniques taken from Michie S, Richardson M, Johnston M. The behavior change technique taxonomy (V1) of 93 hierarchically clustered techniques: building an international consensus for the reporting of behavior change interventions. *Ann Behav Med*. 2013;46. BCT definitions and examples can be accessed on the free online BCT taxonomy training website: <http://www.bct-taxonomy.com/>
