# Supplementary material for: Self-management interventions for skin care in people with a spinal cord injury: part 1—a systematic review of intervention content and effectiveness
Source: Spinal Cord. 2018 May 25;56(9):823–36. doi: 10.1038/s41393-018-0138-3 (PMC6128818; doi:10.1038/s41393-018-0138-3)
Supplement: Supplementary file 3 — List of excluded references at full-text [file 41393_2018_138_MOESM3_ESM.docx]

**Supplementary File 3. List of references excluded during full-text screening**

1) Beck LA, Scroggins LM. Optimizing health of individuals with tetraplegia. *SCI Nurs.* 2001;18(4):181-6.

2) Block P, Vanner EA, Keys CB, Rimmer JH, Skeels SE. Project Shake-It-Up: using health promotion, capacity building and a disability studies framework to increase self efficacy. *Disabil Rehabil.* 2010;32(9):741-54.

3) Carr S, Wilson B. Promotion of pressure-relief exercising in a spinal injury patient: A multiple baseline across settings design. *Behav Psychother.* 1983;11(4):329-36.

4) Duci B, Saperstein I. SCI home care: transitional rehabilitation as a component of follow-up car. *SCI Nurs.* 1986;3(1):6-9.

5) Jones HW. A follow-up service--supplemented by domicillary visits. *Paraplegia.* 1972;10(2):111-4.

6) Pollack SF, Zuger RR, Walsh J. Moving Out Services for Education and Support (MOSES): a model program for individuals with spinal cord injury. *SCI Nurs.* 1992;9(3):79-82.

7) Ravesloot C, Seekins T, White G. Living Well With a Disability Health Promotion Intervention: Improved Health Status for Consumers and Lower Costs for Health Care Policymakers. *Rehabil Psychol.* 2005;50(3):239-45.

8) Rawl SM, Easton KL, Kwiatkowski S, Zemen D, Burczyk B. Effectiveness of a nurse-managed follow-up program for rehabilitation patients after discharge. *Rehabil Nurs*. 1998;23(4):204-9.

9) Steinberg FU. The management of patients with spinal cord injury by a hospital-based home care programme. *Paraplegia.* 1975;12(4):256-50.

10) Weinel D. The Sunshine Network Teleconsultation Initiative for SCI Veterans in Community Settings. *SCI Nurs.* 2000;17(1):22-3.

11) Bailey J, Dijkers MP, Gassaway J, Thomas J, Lingefelt P, Kreider SE, et al. Relationship of nursing education and care management inpatient rehabilitation interventions and patient characteristics to outcomes following spinal cord injury: the SCIRehab project. *J Spinal Cord Med.* 2012;35(6):593-610.

12) Bishop CH, Droste LR. Pressure ulcer prevention in the patient with spinal cord injury on hemodialysis. Nephrol Nurs J 2014;41(1):93-6, 99.

13) Brace JA, Schubart JR. A prospective evaluation of a pressure ulcer prevention and management E-Learning Program for adults with spinal cord injury. *Ostomy Wound Manage*. 2010;56(8):40-50.

14) Chapman K, McGinnis-Rake C, O'Halloran W, Paul S, Virden JA. Convincing the non-compliant patient to change his behavior. *Ostomy Wound Manage*. 1991;35:45-6, 48, 50-1.

15) Deokali M. Prevention of pressure sores in spinal paraplegia and tetraplegia. *SA Nurs J.* 1971;38(4):14-8.

16) Engstrand JL. A nursing challenge: effective patient education. *ARN J*. 1979;4(5):15-8.

17) Jones ML, Matthewson CS, Adkins VK, Ayllon T. Use of behavioral contingencies to promote prevention of recurrent pressure ulcers. *Arch Phys Med Rehabil.* 2003;84(6):796-802.

18) Kennedy P, Evans MJ, Berry C, Mullin J. Comparative analysis of goal achievement during rehabilitation for older and younger adults with spinal cord injury. *Spinal Cord.* 2003;41(1):44-52.

19) Kooijmans H, Post MW, van der Woude LH, de Groot S, Stam HJ, Bussmann JB. Randomized controlled trial of a self-management intervention in persons with spinal cord injury: design of the HABITS (Healthy Active Behavioural Intervention in SCI) study. *Disabil Rehabil.* 2013;35(13):1111-8.

20) Krouskop TA, Noble PC, Garber SL, Spencer WA. The effectiveness of preventive management in reducing the occurrence of pressure sores. *J Rehabil RB.* 1983;20(1):74-83.

21) Malament IR, Dunn ME, Davis R. Pressure sores: an operant conditioning approach to prevention. *Arch Phys Med Rehabil.* 1975;56(4):161-4.

22) Maugham L, Cox R, Amsters D, Battistutta D. Reducing inpatient hospital usage for management of pressure sores after spinal cord lesions. *Int J Rehabil Res.* 2004;27(4):311-5.

23) Rothery FA. Preliminary evaluation of a pressure clinic in a new spinal injuries unit. *Paraplegia.* 1989;27(1):36-40.

24) Bloemen-Vrencken JH, de Witte LP, Post MW, Pons C, van Asbeck FW, van der Woude LH, et al. Comparison of two Dutch follow-up care models for spinal cord-injured patients and their impact on health problems, re-admissions and quality of care. *Clin Rehabil.* 2007;21(11):997-1006.

25) Zemper ED, Tate DG, Roller S, Forchheimer M, Chiodo A, Nelson VS, et al. Assessment of a holistic wellness program for persons with spinal cord injury. *Arch Phys Med Rehabil.* 2003;82(12):957-68.

26) Dinsdale D, Thurber D, Hough E, Rencz S. Community based monitoring for spinal man. *Can J Public Health.* 1981;72(3):195-8.

27) Mackelprang JL, Hoffman JM, Garbaccio C, Bombardier CH. Outcomes and Lessons Learned From a Randomized Controlled Trial to Reduce Health Care Utilization During the First Year After Spinal Cord Injury Rehabilitation: Telephone Counseling Versus Usual Care. *Arch Phys Med Rehabil.* 2016;97(10):1793-6.

28) Patterson RP, Strandal LC. Warning device for the prevention of ischaemic ulcers in patients who have injured the spinal cord. *Med Biol Eng.* 1973;11(4):504-5.

29) Ravesloot C, Seekins T, Young QR. Health promotion for people with chronic illness and physical disabilities: the connection between health psychology and disability prevention. *Clin Psychol Psychother.* 1998;5(2):76-85.

30) Zahl ML, Compton DM, Kim K, Rosenbluth JP. SCI/D forum to increase active living: The effect of a self-efficacy and self-affirmation based SCI/D forum on active living in adults with spinal cord injury/disease. *SCI Psychosoc Process.* 2008;21(2):5-13.

31) Raghaw SS. Efficacy of Peer Counselling in Facilitating Life Long Adjustment of People with SCI in South-East Asian Countries: P-100. *Top Spinal Cord Inj Rehabil.* 2011;16(-1):94-5.

32) Norris WC, Noble CE, Strickland SB. SILS, spinal injiury learning series. Jackson: University Press of Mississippi; 1981.[Note: Not eligible for inclusion in the review but used as intervention materials]

33) Mercier HW, Jette A, Houlihan B. Differential impact and use of a telehealth intervention by persons with multiple sclerosis or spinal cord injury. *Arch Phys Med Rehabil.* 2014;95(10):e34-5.

34) Liungberg I, Libin A, Groah S. Effect of a SCI navigator on pressure ulcer knowledge. *J Spinal Cord Med.* 2010;33(2):183.

35) Libin AK. YouTube as an online disability self-management tool in persons with spinal cord injury. *Top Spinal Cord Inj Rehabil.* 2011;16(3):84-92.

36) Andberg MM, Rudolph A, Anderson TP. Improving skin care through patient and family training. *Top Clin Nurs.* 1983;5(2):45-54.

37) Bliss MR. Spinal injury and pressure sore prevention. *J Wound Care.* 1997;6(10):474.

38) Engel KL, Waring WP. Developing and implementing a transdisciplinary educational model for the prevention of pressure ulcers. *SCI Nurs.* 2008;25(1):31-9.

39) Prevention and treatment of pressure sores in spinal cord injuries. *J Clin Nurs.* 1993;2(3):188.

40) Klose KJ, Needham BM, Schmidt D, Broton JG, Green BA. An assessment of the contribution of electromyographic biofeedback as an adjunct therapy in the physical training of spinal cord injured persons. *Arch Phys Med Rehabil.* 1993;74(5):453-6.

41) Hilgard M, Ritterband L, Baxter K, Alfano A, Ratliff C, Kinzie M, et al. Development and perceived utility and impact of a skin care Internet intervention. *Internet Interv.* 2014;1(3):149-57.

42) Guy S, Kras-Dupuis A, Wolfe D. Implementation of a pressure ulcer prevention education best practice for persons spinal cord injury. *J Spinal Cord Med.* 2014;37(5):617-8.

43) Guihan M, Holmes SA, Bombardier CH, Ehde DM, Rapacki LM. Self-management to prevent ulcers in spinal cord injury. *J Spinal Cord Med.* 2013;36(5):520.

44) Brace J, Schubart J. A prospective evaluation of a pressure ulcer prevention and management e-learning program for adults with spinal cord injury. *J Wound Ostomy Continence Nurs.* 2011;38(4S):S8-9.

45) Bishop CH, Droste LR. Pressure ulcer prevention in the spinal cord injured. *Virginia Nurses Today.* 2014;22(1):12.
