# Supplementary material for: Self-management interventions for skin care in people with a spinal cord injury: part 1—a systematic review of intervention content and effectiveness
Source: Spinal Cord. 2018 May 25;56(9):823–36. doi: 10.1038/s41393-018-0138-3 (PMC6128818; doi:10.1038/s41393-018-0138-3)
Supplement: Supplementary file 4 — Used and missing intervention materials [file 41393_2018_138_MOESM4_ESM.docx]

**Supplementary File 4**. Used and missing intervention materials

| **Reference** |  | **Intervention materials** | | **Identification method or Reason for non-use** |  |
| --- | --- | --- | --- | --- | --- |
| Garber (2002),  Rintala (2008) | Used | U.S. Department of Health and Human Services. Public Health Service. Agency for Health Care Policy and Research (AHCPR). Treating pressure sores: consumer guide, Clinical Practice Guideline No. 15. Publication No. 95-0654. Washington (DC); 1994 Dec. | | Referenced in primary papers; located by reviewers |  |
|  |  | U.S. Department of Health and Human Services, Public Health Service, Agency for Health Care Policy and Research (AHCPR). Preventing pressure ulcers: patient guide. Clinical Practice Guideline No. 3. Publication No. 92-0048. Washington (DC): 1992 May. | | Referenced in primary papers; located by reviewers |  |
|  | Not used | Hammond MC, Umlauf RL, Matteson B, Perduta-F'ulginiti S, editors. Pressure sores. In: Yes, you can! A Guide to self-care for persons with spinal cord injury. 2d ed. Paralyzed Veterans of America: Washington, DC; 1989.p. 67-70. | | Not located via University of Ottawa library services & Authors no longer have copy |  |
|  |  | Ackerman J, Burnett M, Clark B, Garber S, Probst D. A personal guide to healthy skin. The Institute for Rehabilitation and Research and The Rehabilitation Research and Training Center on Community-Oriented Services for Persons with Spinal Cord Injury. Houston, Texas; 1990. | | Not located via University of Ottawa library services & Authors no longer have copy |  |
| Guihan (2014) | Used | PowerPoint slides used to support intervention delivery | | Provided by authors following email contact |  |
|  |  | Table displaying content of intervention | | Provided by authors following email contact |  |
|  |  | Short intervention content description | | Provided by authors following email contact |  |
|  | Not used | Maddox S. Paralysis resource guide. Short Hills: Christopher Reeve Foundation; 2006. | | Not located via University of Ottawa library services & Authors no longer have copy |  |
|  |  | Intervention manual for self-management group sessions | | Authors no longer have copy |  |
| Norris (1982) | Used | Norris W, Strickland, SB, Noble, CE. SILS: Spinal Injury Learning Series. Mississippi Methodist Rehabilitation Center, Jackson: University Press of Mississippi; 1981. | | Located by reviewers during screening process |  |
|  | Not used | No intervention materials known to be missing | | Not applicable |  |
| Houlihan (2013),  Mercier (2015) | Used | CareCall scripts (all modules: skin care, depression and health care utilization) | | Provided by authors following email contact |  |
|  |  | CareCall resource book | | Provided by authors following email contact |  |
|  |  | Pre-recorded audio vignettes (patient advice) | | Provided by authors following email contact |  |
|  | Not used | No intervention materials known to be missing | | Not applicable |  |
| Worobey (2016) | Used | Wheelchair skills training program manual, version 4.2 | | Referenced in primary paper; located by reviewers |  |
|  | Not used | No intervention materials known to be missing | | Not applicable |  |
| Best (2016) | Used | Wheelchair skills training program manual, version 4.1 | | Referenced in primary paper; located by reviewers |  |
|  |  | Sample of participant intervention manual | | Provided by authors following email contact |  |
|  | Not used | Full participant intervention manual | | Author preference to keep confidential |  |
| Ozturk (2011) | Used | Wheelchair skills training program manual, version 4.1 | | Referenced in primary paper; located by reviewers |  |
|  | Not used | No intervention materials known to be missing | | Not applicable |  |
| Rowland (2006) | Used | None | | Not applicable |  |
|  | Not used | Knowledge and behavioural risk assessments + feedback | | Author non-response |  |
| Rottkamp (1976) | Used | None | | Not applicable |  |
|  | Not used | Diagrammed illustration body positions | | Author email contact unavailable |  |
|  |  | Daily positioning schedule for patients | | Author email contact unavailable |  |
| Schopp (2007) | Used | None | | Not applicable |  |
|  | Not used | 8 minute video | | Author non-response |  |
| Hossain (2016) | Used | None | | Not applicable |  |
|  | Not used | Educational booklet for participants | | Author preference to keep confidential |  |
| Scotzin (1990) | Used | None | | Not applicable |  |
|  | Not used | Unknown if any missing | | Author email contact unavailable |  |
| Kennedy (2003); Phillips (1999); Phillips (2001) | Used | None | Not applicable | | |
|  | Not used | Unknown if any missing | Author non-response | | |

Note: Attempts to contact authors for intervention materials were made regardless of whether intervention materials were mentioned in primary paper.
