# Supplementary figures and images for: Self-management interventions for skin care in people with a spinal cord injury: part 1—a systematic review of intervention content and effectiveness
Source: Spinal Cord. 2018 May 25;56(9):823–36. doi: 10.1038/s41393-018-0138-3 (PMC6128818; doi:10.1038/s41393-018-0138-3)

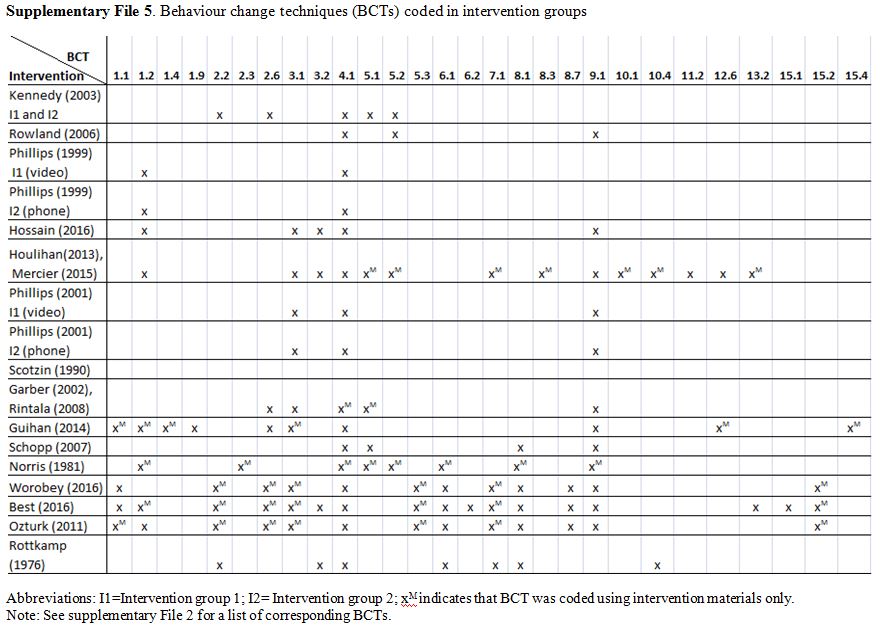

Supplement: Supplementary file 5 — Behavior change techniques coded in intervention groups [file 41393_2018_138_MOESM5_ESM.jpg]

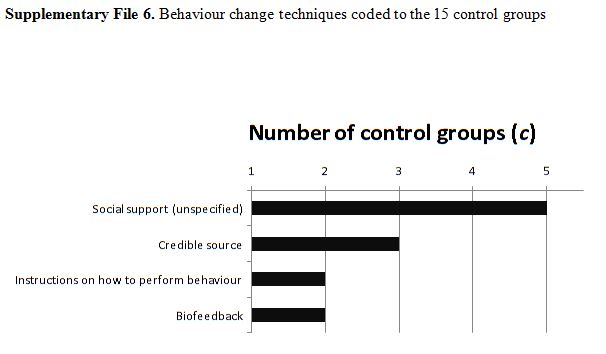

Supplement: Supplementary file 6 — Behavior change techniques coded in control groups [file 41393_2018_138_MOESM6_ESM.jpg]
