# Supplementary material for: Self-management interventions for skin care in people with a spinal cord injury: part 1—a systematic review of intervention content and effectiveness
Source: Spinal Cord. 2018 May 25;56(9):823–36. doi: 10.1038/s41393-018-0138-3 (PMC6128818; doi:10.1038/s41393-018-0138-3)
Supplement: Supplementary file 7 — Risk assessment results for the 10 randomized trials reviewed [file 41393_2018_138_MOESM7_ESM.docx]

**Supplementary File 7.** Risk assessment results for the 10 randomized trials reviewed

| Risk of bias  item  Reference | Random sequence generation | Allocation concealment | Blinding of participants and personnel | Blinding of skin care outcomes | Blinding of pressure ulcer related outcomes | Incomplete outcome data | Selective outcome reporting |
| --- | --- | --- | --- | --- | --- | --- | --- |
| Garber (2002)  Rintala et al (2008) | Low | ? | High | High | High | High | ? |
| Guihan et al (2014) | ? | ? | ? | ? | Low | Low | High |
| Phillips (2001) | ? | ? | ? | NA | ? | ? | ? |
| Houlihan (2013), Mercier (2015) | ? | ? | ? | High | Low | ? | ? |
| Hossain (2013) | Low | Low | High | NA | Low | ? | Low |
| Worobey (2016) | ? | Low | Low | Low | NA | High | High |
| Best (2016) | Low | Low | High | Low | NA | Low | Low |
| Ozturk (2011) | Low | ? | High | High | NA | High | ? |
| Rowland (2006) | ? | ? | ? | Low | ? | ? | ? |
| Rottkamp (1976) | ? | ? | ? | ? | ? | Low | Low |

Notes: High= High risk of bias, low= low risk of bias,? = unclear risk of bias

The ‘unclear’ category was used where there was insufficient detail for a conclusion to be reached, or where there was no published/registered study protocol available (item on selective outcome reporting).

A decision about the selective outcome reporting item was reached by comparing outcomes reported on to measures outlined in registered or published protocols. If a protocol was not available, this item was marked as ‘unclear’. In addition, items on blinding of outcomes were rated ‘not applicable’ if skin care outcomes or pressure-ulcer related outcomes were not measured.
