# Supplementary material for: Self-management interventions for skin care in people with a spinal cord injury: part 1—a systematic review of intervention content and effectiveness
Source: Spinal Cord. 2018 May 25;56(9):823–36. doi: 10.1038/s41393-018-0138-3 (PMC6128818; doi:10.1038/s41393-018-0138-3)
Supplement: Supplementary file 8 — Summary of effectiveness findings for the 10 randomized controlled trials reviewed [file 41393_2018_138_MOESM8_ESM.docx]

**Supplementary File 8**. Summary of effectiveness findings for the 10 randomized controlled trials reviewed

| **Study**  **(first author)** | **Compari-sons**  **(brief description)** | **Risk of bias (number of items)** | **Relevant outcomes measured** | **Results for between group comparisons: Behavioural outcomes (mediators of behaviour and behaviour)** | **Results for between group comparisons: Pressure-ulcer related clinical outcomes** |
| --- | --- | --- | --- | --- | --- |
| Garber (2002),  Rintala (2008) | I: Enhanced education  C: Standard education | High: 4  Low: 1 Unclear: 2 | Knowledge on PUs  (self-report; self-developed)  PU recurrence status at 24 months (self-report)  Time from discharge to recurrence of a PU in pelvic area (self-report) | **% correct knowledge items**:  *Pre-intervention (prior to discharge):*  I (n=20): 54.16 ± 19.18  C (n=21): 54.76 ± 11.93  NS btw groups: t_39_ = -0.12, p < 0.91  *Post-intervention (at discharge):*  I (n=19): 73.86 ± 12.35  C (n=20): 64.00 ±14.74  Sig btw groups: t_37_= 2.26, *p*<0.03  Main effect of time (*F* = 37.23, *p <*0.0001), no main effect of group (*F =* 1.22, *p <* 0.28) but significant time by group effect (*F=* 4.72, *p*< 0.04).  **Effect size (Hedges g with 95% CIs):**  **0.71 (0.06, 1.36)** | **PU recurrence within 24 months**:  I: n=18, C: n=20  Yes – I: n=6 (33.3%); C n=15 (75%)  No – I: n=12 (66.7%) ; C n=5 (25.0%)  Sig btw groups:  *χ*^2^= 8.47, *p*=0.007  **Effect size (Odds Ratio with 95% CIs): 0.17 (0.04, 0.68)**  **Time from discharge to recurrence or end of study (months):**  I (n=18): 19.61 ±7.15  C(n=20): 10.20 ±8.53* |
| Guihan (2014) | I: SM sessions + MI follow-up phone calls  C: Standard SM sessions without skills training component | High:1  Low: 2  Unclear: 4 | Skin care behaviours (self-report; self-developed)  Self-efficacy for PU prevention and management (self-report; validated scale adapted for PUs)  Knowledge on PU (self-report; measure by Garber et al. (2002)  Number of days on bedrest resulting from skin problems (self-report)  Skin status (rated by HCPs using photos taken by patients/carers + medical records)  Time to skin worsening (0-3, 3-6 months after discharge) (self-report)  VA and non-VA skin-related health visits and hospitalizations (self-report; self-developed items) | **% of skin care behaviours performed**:  *Pre-intervention (at admission):*  C (n=72): 74.1± 18.6  I (n=71): 73.8±23.3  Fisher exact test: *p*=0 .92  *3 months post-discharge*:  C (n=72): 79.5 ± 19.6  I (n=71): 83.5 ± 17.5  Fisher exact test: *p*= 0.21  *6 months post-discharge*:  C (n=72): 83.0 ± 14.6  I (n=71): 85.0 ± 15.2  Fisher Exact test: *p*=0.41  **Effect size (Hedges g with 95% CIs):**  **0.13 (-0.19, 0.46)**  % change in skin care behaviors  *0-3 months*:  I (n=71): 9.7± 19.3  C (n=72): 5.4± 22.9  t-test or Wilcoxon test: *p*=0.23  *0-6 months:*  C (n=72): 8.9 ± 18.1  I (n=71): 11.3 ± 20.0  t-test or Wilcoxon test: *p*= 0.45  Self-efficacy – data/statistical tests NR.  Knowledge - data/statistical tests NR. | **Skin worsening during study (%)** (includes worsening of pressure ulcers from baseline, and new wounds):  Yes – C (n=72): 39 (54.2%)  I (n=71): 35 (49.3%)  No – C (n=72): 33 (45.8%)  I (n=71): 36 (50.7%)  Fisher exact test: *p*=0.51  **Effect size (Odds Ratio with 95% CIs): 0.82 (0.43, 1.59)**  **Time to skin worsening (n,%):**  *0-3 months:* C (n=72): 28 (38.9); I (n=71): 26 (36.6%)  *4-6 months*: C (n=72): 11(15.3%);  I (n=71): 9( 12.7%)  Fisher exact test: *p*= 0.86  Number of days on bed rest- data/statistical tests NR.  **VA and non-VA skin-related health care utilization**: NS btw groups (no data/stat tests reported). |
| Phillips (2001) | I: Video and phone based telehealth  C: Standard care | Low: 1  Unclear: 6  NA: 1 | PU prevalence (self-report) | NA | **PU prevalence during one year follow-up:**  No data/stat tests reported. |
| Houlihan (2013),  Mercier (2015) | I: Interactive voice response telehealth system  C: Standard care | High: 1  Low: 1  Unclear: 5 | Skin care behaviours^€^ (self-report; self-developed items; 0-4 scale; greater scores= better compliance)  Presence and severity of PUs (validated PUSH scale applied by nurse during physical exam; higher scores = more severe tissue damage) | **Skin care behaviours (total scale scores - see single item scores below)**^€^**:**  *Pre-intervention:*  C(n=71):18.9±3.6;I(n=71):19.4±3.32  *At 6 months:*  C(n=66): 19.8±2.9; I (n=64): 21.4±2.4  **Effect size (Hedges with 95% CIs):**  **0.60(0.25, 0.95)**  **Skin care behaviours- single items:**  **Skin checks^€^ (1 item):**  *Pre-intervention*:  C(n=71):3.1 ± 1.1; I(n=71):3.1 ±1.1  *At 6 months*:  C (n=66):3.2± 1.0; I(n=65):3.5±0. 9  **Pressure reliefs sitting ^€^ (1 item)**  *Pre-intervention*:  C (n=71):2.8 ±1.0;I(n=71): 2.8 ±1.1  *At 6 months*:  C(n=66):3.0 ± 1.0;I(n=65):3.4 ± 0.9  **Pressure relief in bed^€^ (1 item):**  *Pre-intervention*:  C(n=71): 2.8±1.2;I (n=71):2.8 ± 1.3  *At 6 months*:  C (n=66):2.7± 1.2;I(n=65): 3.4± 1.0  Reported in discussion: The odds of men reporting turning in bed every 2–4 h were 4.5 times higher than for women (*p*=0.008) – no further details provided.  **Wheelchair cushion check^€^ (1 item)**  *Pre-intervention:*  C(n=71):3.4 ± 0.9; I(n=71):3.4± 0.9  *At 6 months*:  C(n=66):3.6 ± 0.8; I(n=65):3.6± 0.8  **Help seeking for skin breakdown^€^ (1 item):**  *Pre-intervention:*  C (n=71):3.0 ± 1.2;I(n=71): 3.3±1.2  *At 6 months:*  C (n=66):3.3 ± 1.0; I(n=65):3.5±0.9  **Keeping skin clean and dry^€^ (1 item)**  *Pre-intervention:*  C (n=71):3.9 ± 0.4;I(n=71):3.9 ±0.3  *At 6 months:*  C (n=66): 4.0 ± 0.0;I(n=65):4.0±0.2 | **PU prevalence (%)**  *Pre-intervention*:  Yes – C (n=71): 15 (21.1%)  I (n=71): 13 (18.3%)  No – C (n=71): 56 (78.9%)  I (n=71): 58 (81.7%)  *At 6 months*^€^*:*  Yes – C (n=66): 10 (15.2%)  I (n=65): 8 (12.3%)  No – C (n=66): 56 (84.8%)  I (n=65): 57 (87.7%)  Logistic regression: no significant effect on development of one or more PUs at 6 months (no stat test data reported).  **Effect size (Odds Ratio with 95% CIs):** **0.79 (0.29, 2.14)**    *A posteriori* analyses on PU prevalence by gender (%):  *Pre-intervention*:  Female: I (n=71): 19%; C (n=71): 21%  Male: I (n=71): 18%; C (n=71): 19%  *At 6 months (n=131)*^€^*:*  Female (n=53): I (n=20): 0(0%);  C (n=33): 6 (18%)  Male (n=78): I (n=45): 8 (18%);  C (n=33): 4 (12%)  One sided test (unclear) indicated that among women, there was a significant group difference in the % with one or more PUs (*p*=0.04). No positive intervention effect for men (*p*>0.05).  **PUSH (score)****  *At baseline* (whole sample):  C (n=71): 2.2 ± 6.31  I (n=71): 1.48 ± 4.18  For SCI-subsample specifically:  C (n=53): 2.0 ± 6.2  I (n=53): 1.3 ± 3.2  *At 6 months (whole sample):*  C (n=71): 1.83 ± 5.61  I (n=71): 1.49 ± 5.6  For SCI-subsample specifically:  C (n=53): 1.7 ± 5.5  I (n=53): 2.0 ± 6.4  No stat tests run for the above data.  **Effect size (Hedges g with 95% CIs) calculated using SCI sub-sample data: 0.05 (-0.33, 0.43)** |
| Hossain (2016) | I: Phone-based telehealth for monitoring and support  C: Standard care | High: 1  Low: 4  Unclear: 1  NA: 1 | Presence and severity of PUs (validated PUSH  scale applied by HCP during physical exam) | NA | **PU prevalence**  *Pre-intervention*:  C (n=15): 0 (0%); I (n=15): 0 (0%)  *At 2 years*:  C (n=14): 2 (14.3%); I (n=14): 3 (21.4%)  Note: 1 death in intervention group caused by PU (in addition to the above 3 with a PU).  **Effect size (Odds Ratio with 95% CIs): 2.40 (0.36, 15.94)**  PUSH score (only people with a PU):  *Baseline:* NA (n=0 with a PU)  *At 2 years*:  C (n=2):13.0 ± 5.7; I (n=3): 10.7 ±1.2  (Note: deceased with PU not included) |
| Worobey (2016) | I: Wheelchair skills training - participants identified a selection of skills to work on  C: No training | High: 2  Low: 3  Unclear:1  NA: 1 | Skills: Wheelchair Skills Test Questionnaire (WST-Q, validated tool) – assessment of 32 individual skills including pressure relief skills (administered by trainers). | WST-Q scores measured at 1 month follow-up and expressed as a summary score; individual pressure relief skill scores NR.  Note: Data provided shows that none of the intervention participants chose to focus on improving pressure relief skills during the intervention. | NA |
| Best (2016) | I: Wheelchair skills training –participants identified up to 5 skills to work on  C: no training | High: 1  Low: 5  NA: 1 | Skills: Wheelchair Skills Test Questionnaire (WST-Q) including pressure relief - see above. | WST-Q scores expressed as a summary score; individual pressure relief skill scores NR.  Note: Data shows that none of the intervention participants chose to improve pressure relief skills during the intervention. | NA |
| Ozturk (2011) | I: Wheelchair skills training –participants focused on wheelchair skills not successfully completed at baseline  C: no training | High:3  Low: 1  Unclear:2  NA: 1 | Skills: Wheelchair Skills Test Questionnaire (WST-Q) including pressure relief- see above. | WST-Q scores expressed as a summary score; individual wheelchair skill data shown but only for those skills for which baseline and follow-up scores changed by more than 25% (does not include pressure relief skills).  Note: 100% intervention participants successfully and safely completed pressure relief skills at baseline therefore none worked on these skills during the intervention (personal communication with authors). | NA |
| Rowland (2006) | I: Risk assessment  (knowledge, behaviours)  of secondary complica-tions (including PUs), with automated + in person feedback  C: Risk assessment of secondary complica-tions, without feedback | Low: 1  Unclear: 6 | Knowledge on prevention of 5 secondary (including PUs) (self-developed; self-report)  Adherence to preventive behaviours, including skin care (self-developed; self-report)  Development of 5 secondary conditions (self-report + medical record verification) and time to development | **% correct responses for PU items:**  *Pre-intervention*:  I (n=NR): 82.3 (SDs NR)  C (n=NR): 84.0 (SDs NR)  Difference btw groups at baseline approached significance.  *At one year*:  I (n=NR): 86.9 (SDs NR)  C (n= NR): 84.2 (SDs NR)  No stat tests run for btw group differences .  Adherence to preventive behaviours including skin care- no data/stat tests reported. | **PU prevalence**  *At baseline:* Data not clearly reported  *During 1 year study period:*  I (n=NR): 23.7%  C (n=NR): 27.6%  *χ*^2^(1, *N*=67)=0.132, *p*=0.72  NS btw groups in time to development (no data/stat test results reported). |
| Rottkamp (1976) | I: Inpatient body positioning training program  C: Standard body positioning practices in ward | Low: 2  Unclear: 5 | Frequency of change of body position (HCP observations)  Frequency of patient-initiated change of body position (HCP observations)  Frequency of face lying in a 24h period (HCP observations)  Length of intervals of prolonged skin pressure (HCP observations)  Skills: Degree of assistance required for change of body position (self-developed scale; completed by HCP; higher score: more assistance)  PU lesions (examinations by HCP) | **Skin care behaviours**  **Change from baseline in frequency of changes in body position*****:  I (n=5): +3 ± 1.7  C (n=5): -0.4 ± 1.5  Significantly greater change for intervention group (U=2, *p*=0.016).  **Change from baseline in frequency of patient-initiated changes of body position*****:  I (n=5): +4 ± 1  C (n=5): +0.6 ±0.9  Significantly greater change for intervention group (U=0, *p*= 0.004).  **Change from baseline in frequency of face lying*****:  I (n=5): 0.8 ± 0.4  C (n=5): 0.2 ± 0.4  NS btw groups (Fischer test, no test results provided).  **Change from baseline in frequency of intervals of prolonged skin pressure*****:  I (n=5): -2 ± 1.4  C (n=5): 0.6 ± 1.3  Significantly greater decrease for intervention group (U=0, *p*=0.004).  **Change from baseline in degree of assistance required to change position*****:  I (n=5): -3 ± 1.2  C (n=5): -1.6 ±1.0  Significantly greater change for intervention group (U=4, *p*=0.048). | **PU prevalence**:  *Pre-intervention*:  I (n=5): 4/5  C (n=5): 0/5  *At 4 weeks:*  I (n=5): Baseline PU status unchanged for 2 participants but no data provided on the 2 remaining participants with a PU at baseline.  C (n=5): 2  Pre and post treatment observations indicated a change in the status of pressure lesions in study patients (no further information provided). |

Abbreviations: btw=between; CI= confidence intervals; HCP: health care professional; MI= motivational interviewing; NA=not applicable; NR= not reported; PU= pressure ulcer; sig= significant; SM= self-management; VA= Veterans Affairs.

*Calculated using mean and SD values provided in Rintala et al. (2008) in which the control group is split into two sub-groups (according to data collection differences, rather than active ingredients received which are identical).

**Calculated by means and SD values provided in Mercier et al. (2015).

^€^Data sent by authors. In Houlihan (2013) & Mercier (2015), summary skin care scale scores were calculated by reviewers, as well as individual item scores.

***Calculated using the individual participant scores provided in Rottkamp et al. (1976) that are presented in graphical format.

Notes: Effect sizes (Hedges g for continuous data and Odds Ratio for categorical data) were calculated for studies where the total sample exceeded 10 participants, and where complete data were available. They were calculated for the following variables: mediators of skin care, skin care behaviours, and PU prevalence and severity (*i.e.* did not include time to recurrence or skin worsening). When both mean scores at follow-up and percentage change scores from baseline were reported by authors (Guihan et al., 2014), effect size calculations were made using mean scores at follow-up. When scale and item level data were available (Houlihan [2013] & Mercier [2015]), effect sizes were calculated using summary scale scores. When complete data were reported for an SCI sub-sample (Houlihan [2013] & Mercier [2015]), these were used for effect size calculations.
